# Targeted hypermutation of putative antigen sensors in multicellular bacteria

**DOI:** 10.1073/pnas.2316469121

**Published:** 2024-02-14

**Authors:** H. Doré, A. R. Eisenberg, E. N. Junkins, G. E. Leventhal, Anakha Ganesh, O. X. Cordero, B. G. Paul, D. L. Valentine, M. A. O’Malley, E. G. Wilbanks

**Affiliations:** ^a^Department of Ecology, Evolution and Marine Biology, University of California, Santa Barbara, CA 93106; ^b^Department of Chemical Engineering, University of California, Santa Barbara, CA 93106; ^c^Department of Civil and Environmental Engineering, Massachusetts Institute of Technology, Cambridge, MA 02139; ^d^Bay Paul Center, Marine Biological Laboratory, Woods Hole, MA 02543; ^e^Department of Earth Science, University of California, Santa Barbara, CA 93106; ^f^Marine Science Institute, University of California, Santa Barbara, CA 93106; ^g^Department of Bioengineering, University of California, Santa Barbara, CA 93106; ^h^Department of Bioengineering, University of California, Santa Barbara, CA 93106

**Keywords:** microbial ecology, targeted mutation, diversity-generating retroelements, multicellularity, bacterial immune systems

## Abstract

Diversity-generating retroelements (DGRs) are used by bacteria, archaea, and viruses as a targeted mutagenesis tool. Through error-prone reverse transcription, DGRs introduce random mutations at specific genomic loci, enabling rapid evolution of these targeted genes. However, the function and benefits of DGR-diversified proteins in cellular hosts remain elusive. We find that 82% of DGRs from one of the major monophyletic lineages of DGR reverse transcriptases are encoded by multicellular bacteria, which often have two or more DGR loci in their genomes. Using the multicellular purple sulfur bacterium *Thiohalocapsa* sp. PB-PSB1 as an example, we characterized nine distinct DGR loci capable of generating 10^282^ different combinations of target proteins. With environmental metagenomes from individual *Thiohalocapsa* aggregates, we show that most of PB-PSB1’s DGR target genes are diversified across its biogeographic range, with spatial heterogeneity in the diversity of each locus. In *Thiohalocapsa* PB-PSB1 and other bacteria hosting this lineage of cellular DGRs, the diversified target genes are associated with NACHT-domain anti-phage defenses and putative ternary conflict systems previously shown to be enriched in multicellular bacteria. We propose that these DGR-diversified targets act as antigen sensors that confer a form of adaptive immunity to their multicellular consortia, though this remains to be experimentally tested. These findings could have implications for understanding the evolution of multicellularity, as the NACHT-domain anti-phage systems and ternary systems share both domain homology and conceptual similarities with the innate immune and programmed cell death pathways of plants and metazoans.

## Significance

To defend themselves against pathogens, bacteria employ a wide range of conflict systems, some of which are enriched in multicellular bacteria. Here, we show that numerous multicellular bacteria use related diversity-generating retroelements (DGRs) to diversify such putative conflict systems. Error-prone reverse transcription in DGRs introduces random, targeted mutations and rapid diversification. We used *Thiohalocapsa* PB-PSB1, a member of multicellular bacterial consortia, to study this association between conflict systems and DGRs. We characterized the natural diversity of PB-PSB1 DGRs and propose they function as hypervariable antigen sensors. If their role in pathogen defense is confirmed, accumulation of these DGR-diversified systems in multicellular bacteria would suggest that rapidly diversifying immune systems confer important fitness advantages for the evolution of multicellularity.

In the evolution of life, a handful of major transitions mark turning points in the emergence of complexity (1, 2). Such evolutionary transitions, including from genes to genomes and from single cells to multicellular organisms, represent a shift in the nature of the individual and require cooperation among previously distinct entities. Explaining the emergence of cooperation remains a major challenge in understanding these transitions. Why should a cell sacrifice its individual interests in favor of the collective? Kin selection and inclusive fitness are commonly invoked to explain the evolution of cooperation: both theory and empirical evidence indicate that the clonality of the group is critical to minimizing conflict and paving the way for multicellularity (3, 4). However, close physical association in a group with few genetic differences also creates conditions for an infectious epidemic. In formulating his social evolutionary theory, Hamilton recognized that, given this lack of genetic diversity, disease could represent a major constraint on the emergence of multicellularity (5). How, then, do nascent multicellular forms balance the risks of infection with the benefits of cooperation?

While innate immunity was classically thought to have emerged among multicellular metazoans, the discovery of many novel defense systems has revealed the bacterial origins of numerous key components of innate immunity (6–11). Intriguingly, several of these recently discovered putative defense systems were found to be particularly enriched in multicellular bacteria (8, 12–14), defined here as bacteria forming multicellular structures through cell–cell adhesion, where close relatives engage in coordinated activities (15). These defense systems in multicellular bacteria were hypothesized to mediate immune-like recognition of invaders and trigger programmed cell death. As described for characterized bacterial abortive infection mechanisms, an infected cell’s premature death would stop phage epidemics by preventing the replication and release of phages near the cell’s clonal kin. Protein- and carbohydrate-binding domains were proposed to act as sensors of an invading phage, while effector domains commonly included trypsin- or caspase-type peptidases. In some of these systems, short non-enzymatic adapter domains fused to sensor and effector domains (“effector associated domains” or EADs) are thought to mediate the assembly of a protein complex akin to the eukaryotic apoptosome and inflammasome, and in some cases are homologous to eukaryotic domains with this function (e.g., death-like and TRADD-N domains) (13). The formation of these protein complexes, and the resulting programmed cell death, is thought to be tightly regulated by the activity of associated NTPase or protein kinase domains (8, 16).

**Fig. 1.**
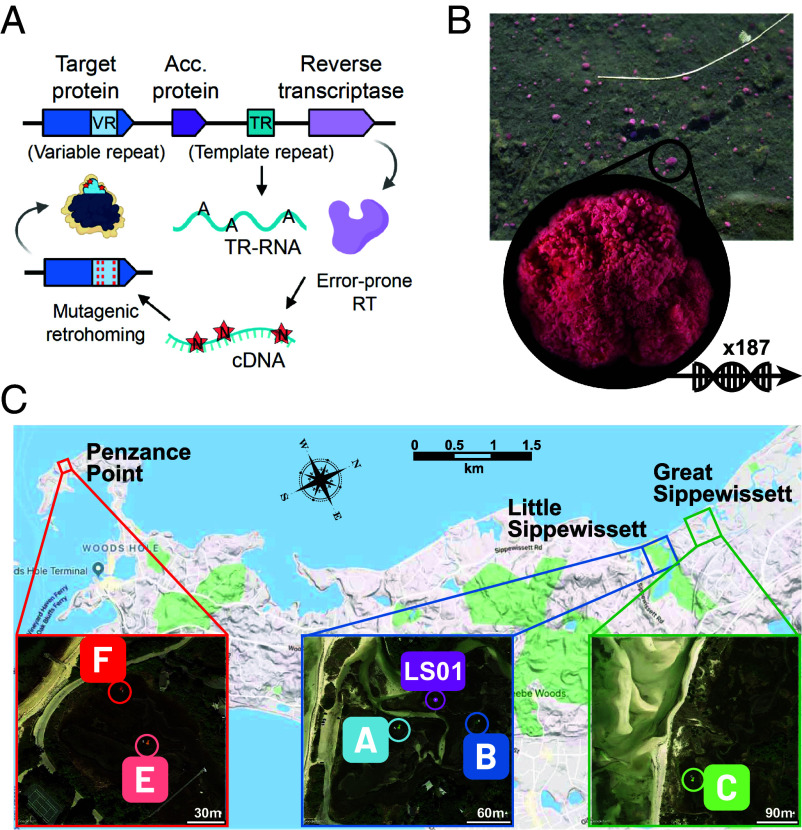
Schematic of DGR mechanism and sampling locations for the “pink berry” bacterial consortia. (*A*) Mutagenic retrohoming introduces mutations within the variable repeat (VR, light blue) of a target protein (dark blue). The error-prone reverse transcriptase (RT, light purple), which complexes with accessory protein(s) (acc, dark purple), introduces random nucleotides at adenine positions in the template repeat (TR, teal) generating a hypervariable TR cDNA that recombines into the VR of the target. (*B*) Individual pink berry consortia were sampled in three salt marshes (Little Sippewissett, LS; Great Sippewissett, GS and Penzance Point, PP) and sequenced with both long read (n = 3, site LS01) and short read (n = 184, sites A, B, C, E, and F) technologies at six sites across their geographic range (*C*).

In several studies, these novel systems were found to be associated with diversity-generating retroelements (DGRs) (8, 13) or with the ligand binding domain DGRs commonly diversify (14). Discovered twenty years ago, DGRs are a unique class of retroelements found in bacteria, archaea, and viruses that generate massive sequence variation at specific protein-coding regions by mutagenic retrohoming (17–21). The central component of the DGR mechanism is an error-prone reverse transcriptase, which incorporates random nucleotides at adenine sites when reverse transcribing a small RNA molecule (the template repeat, TR) (22). This mutated TR-cDNA then specifically recombines into a homologous region in the adjacent target gene (the variable repeat, VR; Fig. 1*A*). This process mutates the target gene at specific positions in the VR that correspond to adenines in the TR. The TR DNA sequence itself remains unaltered, enabling repeated rounds of diversification. The VR is most often located within a C-type lectin (CLec) fold (23–25), a ligand binding domain that can accommodate high amino acid diversity while maintaining protein stability (23, 26).

While much of the DGR mechanism has been elucidated, the function and benefit of DGRs for bacteria and archaea remain mysterious. Since the discovery of the first DGR as a mechanism for tropism switching in *Bordetella* bacteriophage (17, 18), very few DGR targets have been functionally characterized. This is particularly true for cellular (i.e., non-viral) DGRs: DGR diversification has been molecularly confirmed only in *Legionella pneumophila* (27), and the molecular structure was determined for a single cellular DGR target protein from *Treponema denticola* (24). In both cases, the target genes are outer membrane lipoproteins thought to diversify the cell surface of these known pathogens. All other DGR cellular targets have been only computationally predicted. Based on protein homology, diverse functions have been predicted for these target proteins, from host interactions (20, 28, 29) to signal transduction (20, 30, 31) to antiviral defense (21, 29, 32, 33). In multicellular cyanobacteria, Vallota-Eastman et al. argued that DGR targets were likely involved in signal transduction to mediate unknown responses potentially including regulation of cellular homeostasis, cellular differentiation, or programmed cell death (31). Hence, the function of DGR target proteins, particularly for non-pathogenic microbes, remains elusive (21, 25).

The association of novel defense systems with DGRs in multicellular bacteria suggests an intriguing possibility: the diversification of antigen sensors, analogous to the somatic hypermutation of the vertebrate adaptive immune system (34). Here, we explore this possibility using multispecific bacterial consortia, the “pink berries” from salt marshes near Woods Hole, MA (USA), where the most abundant species was reported to have this association between DGRs and predicted conflict systems in two previous studies (8, 13). These millimeter-sized aggregates, found at the water-sediment interface (Fig. 1*B*), offer a model to study bacterial multicellularity across time and space (35). They have a relatively simple species composition, with a few species accounting for most of the cells (36). The dominant species is *Thiohalocapsa* PB-PSB1, a purple sulfur bacterium that grows in dense cellular clumps embedded in an exopolymer matrix and makes up more than half the cells and the majority of the biovolume (35, 36). *Thiohalocapsa* PB-PSB1 is closely associated with a symbiotic species of sulfate-reducing bacteria (PB-SRB1), which catalyzes a cryptic sulfur cycle within the consortia (36). Although these bacteria remain uncultivated, the genome of PB-PSB1 has recently been assembled into a single circular contig from long-read metagenomes, revealing its large size (8 Mb) and enrichment with transposable elements (37). This complete genome allows for a detailed exploration of DGR systems and their natural variation in an uncultivated multicellular bacterium.

We find that the multicellular bacterium *Thiohalocapsa* PB-PSB1 hypermutates putative antigen sensors using a suite of distinct DGR loci. Its genome encodes an unusually high number of distinctly organized DGR loci that are diversified across PB-PSB1’s geographic range. These DGRs belong to one of two major monophyletic lineages of DGR reverse transcriptases from bacteria and archaea [clade 5 *sensu* (21)], and our in-depth analysis indicates that over 82% of the DGRs in this clade are encoded by multicellular bacteria. We show how mobile genetic elements are involved in the dynamics of DGR loci in PB-PSB1 and use metagenomic data from 187 independent aggregates sampled at six sites across the pink berries’ geographic range to demonstrate that eight DGR loci are diversifying in natural conditions (Fig. 1 *B* and *C*). Our analysis of the genomic context of clade 5 DGRs leads us to propose that they diversify the sensors of bacterial immune systems, and we discuss implications of this function in light of selective pressures acting on multicellular life forms.

## Results

### Detection of Nine DGR in *Thiohalocapsa* PB-PSB1.

**Fig. 2.**
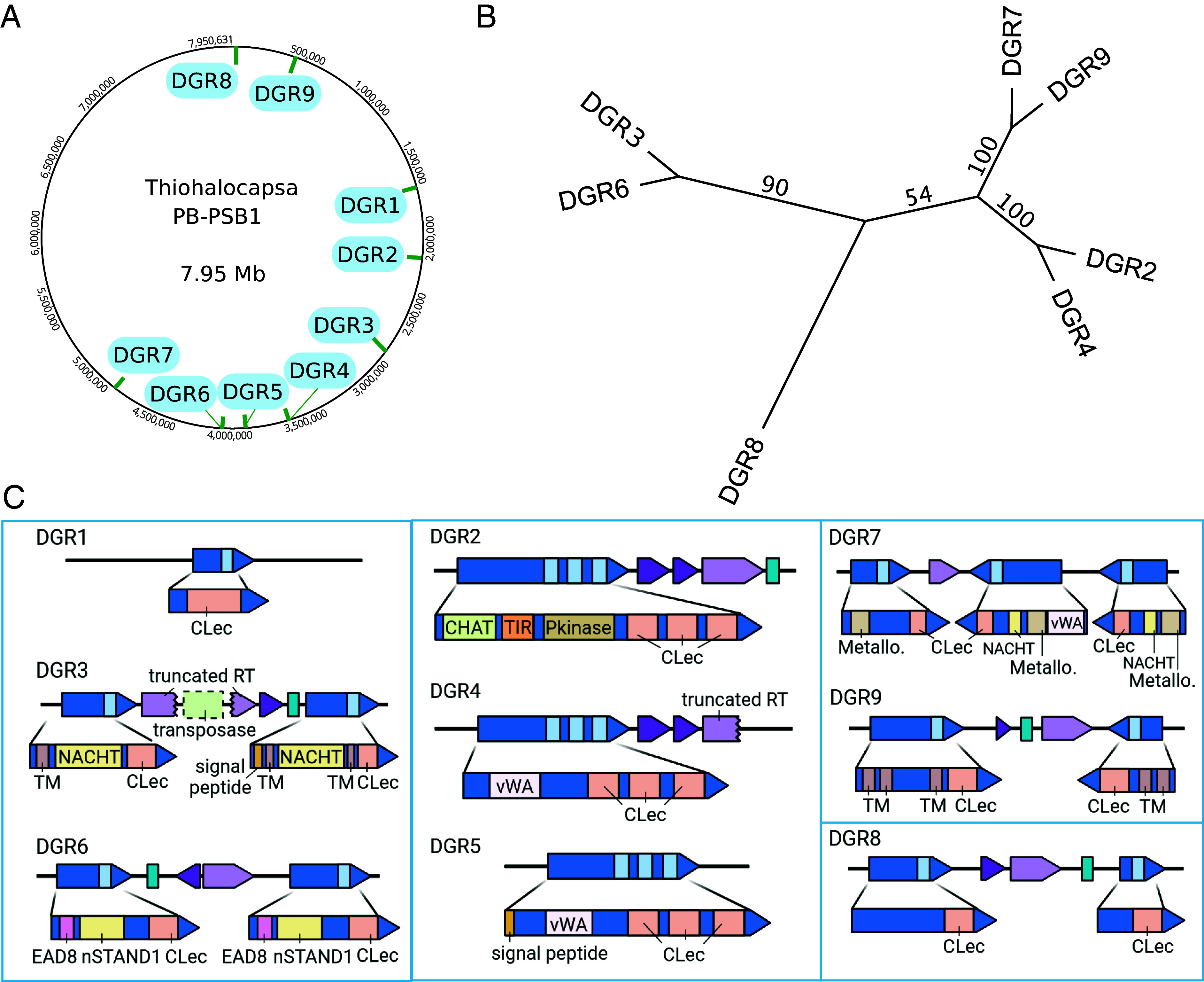
*Thiohalocapsa* PB-PSB1 has nine DGR loci spread across its 7.95 Mb circular, metagenome-assembled genome (*A*). Loci were categorized according to the maximum likelihood phylogeny of their reverse transcriptase genes (RaxML v8.2.11, BLOSUM62+gamma; 100 rapid bootstraps) (*B*), and matching template-variable repeats. The four distinct classes are indicated by blue boxes in (*C*) where each DGR locus is shown with annotated domains for target proteins. Colors scheme corresponds to Fig. 1*A*. Domain abbreviations include: C-terminal lectin domain (CLec); transmembrane domain (TM); von Willebrand domain (vWA); NACHT domain (PF05729); nSTAND1 domain (nSTAND1); CHAT domain (PF12770); Toll/interleukin-1 receptor domain (TIR, PF13676); protein kinase domain (Pkinase; PF00069); 3′,5′-cyclic AMP phosphodiesterase (Metallo; PF00149).

Nine DGR loci were found throughout the genome of *Thiohalocapsa* PB-PSB1, with a total of 15 identified target genes (Fig. 2 and Dataset S1). These nine loci were grouped into four distinct classes based on both the phylogenetic relationships of the DGR reverse transcriptase (RT) genes and the alignment of template and variable repeat regions (TR-VR) (Fig. 2*B* and *SI Appendix*, Fig. S1). Each class includes a single locus with the complete repertoire of functional components (a reverse transcriptase, a template repeat, and an accessory protein), in addition to “degenerate” loci where key machinery is either missing or pseudogenized (Fig. 2).

Together, the 15 DGR target proteins comprise 21 VRs with 521 potential variable positions that affect 325 different codons. The variable positions were almost exclusively found in the codon’s first or second position (often both), a pattern that maximizes the number of possible protein sequences (*SI Appendix*, Fig. S1 and Dataset S2). As was noted in other organisms, the composition of targeted codons also prevents the adenine-directed variation from creating nonsense codons (*SI Appendix*, *Extended Results* and Dataset S2) (23). In PB-PSB1, DGR mutagenesis can yield from 10^9^ to 10^16^ different polypeptides per VR, and up to 2 × 10^45^ different polypeptides per target (Dataset S2). This gives a total of 1.5 × 10^294^ possible sequence combinations at the protein level when accounting for all targets in the genome.

### Multicellular Bacteria Encode Multiple Phylogenetically Related DGR Reverse Transcriptases.

**Fig. 3.**
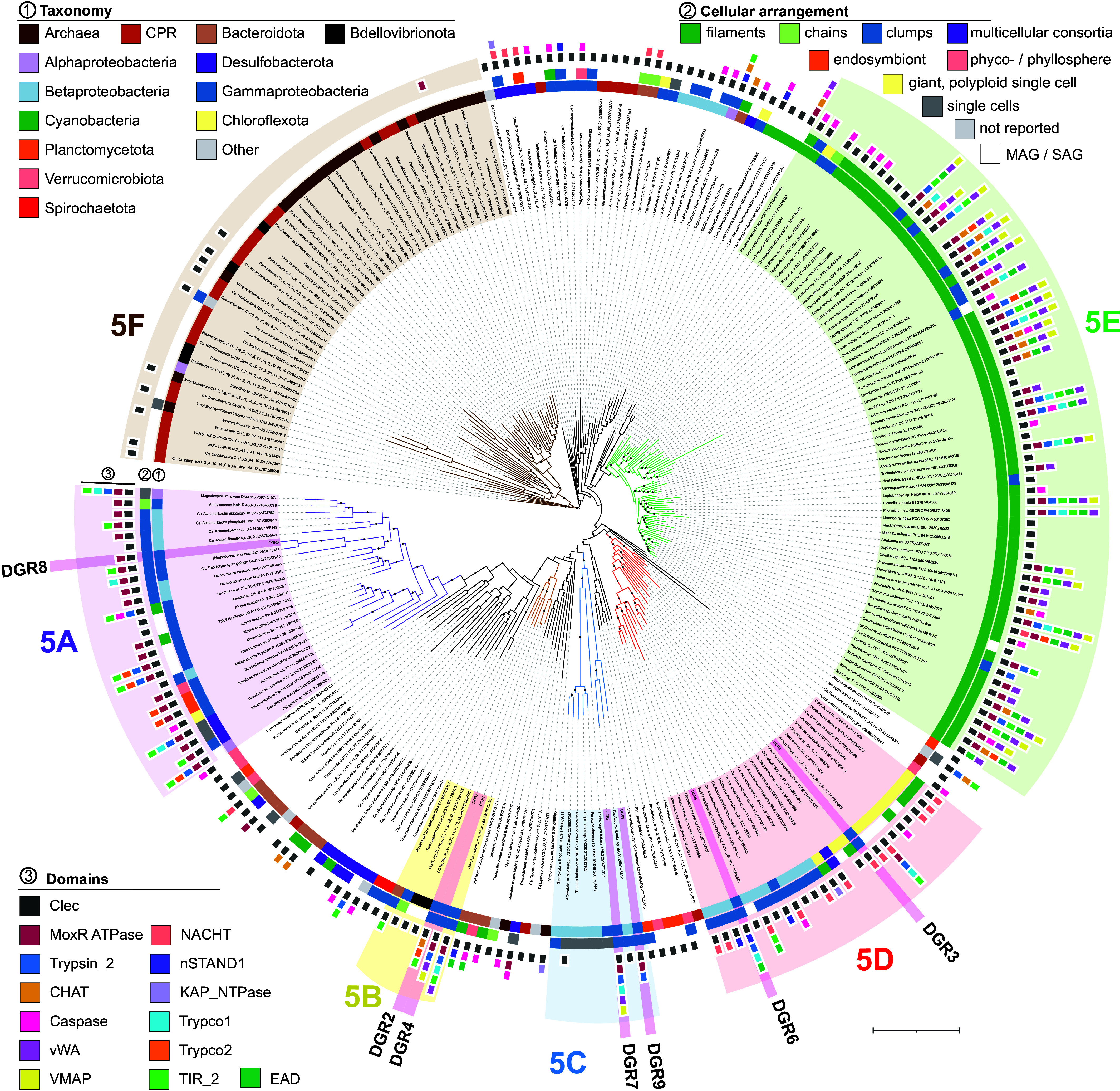
DGR reverse transcriptases (RT) from *Thiohalocapsa* PB-PSB1 are monophyletic with RTs from other multicellular bacteria and syntenic with conflict system domains associated with programmed cell death. The maximum likelihood phylogeny includes all clade 5 RT proteins from ref. 21, excluding metagenomic and SAG sequences not found in the IMG database [IQ-Tree v1.5.5 (38) built-in model selection: Q.pfam+F+R10; 1,000 bootstraps]. The three layers of annotations indicate (from the inside to the outside): 1) the organism taxonomy, 2) the type of cellular arrangement, and 3) the domains of interest detected within 20 kb of the RT. Domains abbreviations and hits in each organism are provided in Dataset S6. The branches shaded in pink correspond to PB-PSB1’s DGR RTs. Bootstrap support greater than 70% is indicated with closed circles (n = 1,000). Scale bar: 1 substitution per site.

The DGR reverse transcriptase genes from PB-PSB1 all belong to one of the two major monophyletic groups of DGR RTs from bacteria and archaea (as opposed to phage), which Roux et al. designated as clade 5 (21). Exploring the phylogeny of clade 5 RTs, we found that 82% of the sequences from described species came from distantly related multicellular or aggregate-forming bacteria (128 of 156, Fig. 3 and Dataset S3). 34 bacteria in this tree encoded multiple DGRs, accounting for 30% of all the sequences we examined (78 of 257 clade 5 RT genes, *SI Appendix*, Table S1). All of the organisms with multiple DGRs are bacteria with multicellular lifestyles, except for the highly polyploid giant bacterium *Achromatium*, and bacteria of the Candidate Phyla Radiation (CPR) and DPANN Archaea, whose morphology remains undetermined (*SI Appendix*, Table S1). CPR and DPANN RTs in clade 5 form a distant monophyletic group (clade 5F, Fig. 3) and are close to the viral-encoded clade 6 RTs (21). Cyanobacterial RTs also form a previously described monophyletic clade (5E, Fig. 3 and *SI Appendix*, Table S1) (31), which we found was almost exclusively multicellular (98%, 63 of 64 characterized species). While duplicate cyanobacterial RTs were typically closely related, multicellular *Proteobacteria, Chlorobi,* and *Verrucomicrobiota* encoded more divergent RTs (Fig. 3 and *SI Appendix*, Table S1).

*Thiohalocapsa* PB-PSB1’s seven error-prone RTs fall in four well-supported clades (5A-5D; Fig. 3) in association with DGR RT sequences from distantly related multicellular bacteria. The organisms in these clades exhibit considerable phylogenetic and morphological diversity (Fig. 3, *SI Appendix*, *Extended Results* and Table S1, and Dataset S3). Multicellular forms range from the obligatory multicellular magnetotactic bacteria (39), to filamentous chloroflexi and planctomycetes (40–42), to mat- and aggregate-forming purple sulfur bacteria (43–46). Other notable organisms with multiple, related DGRs include *Betaproteobacteria* that grow as dense microcolonies in industrially important biofilms, such as *Accumulibacter* species from wastewater treatment reactors and *Nitrosomonas* species from marine biofiltration systems (47–49).

Overall, we find that the RTs forming one of the major cellular lineages of DGRs are found in distantly related multicellular bacteria, many of which contain multiple distinct DGR loci like our model organism, *Thiohalocapsa* sp. PB-PSB1. The divergence among PB-PSB1’s different RTs, and their similarity to RTs of distantly related bacteria, suggests that PB-PSB1 DGRs were acquired through multiple, independent horizontal gene transfer events (Fig. 3 and *SI Appendix*, Table S1). The diversity of multicellular species encoding clade 5 DGRs and the scarcity of planktonic, single-celled species in this clade are notable and suggest that these DGRs may confer benefits specific to multicellular lifestyles.

### Transposons Shape the Evolution of *Thiohalocapsa* PB-PSB1 DGR Loci.

**Fig. 4.**
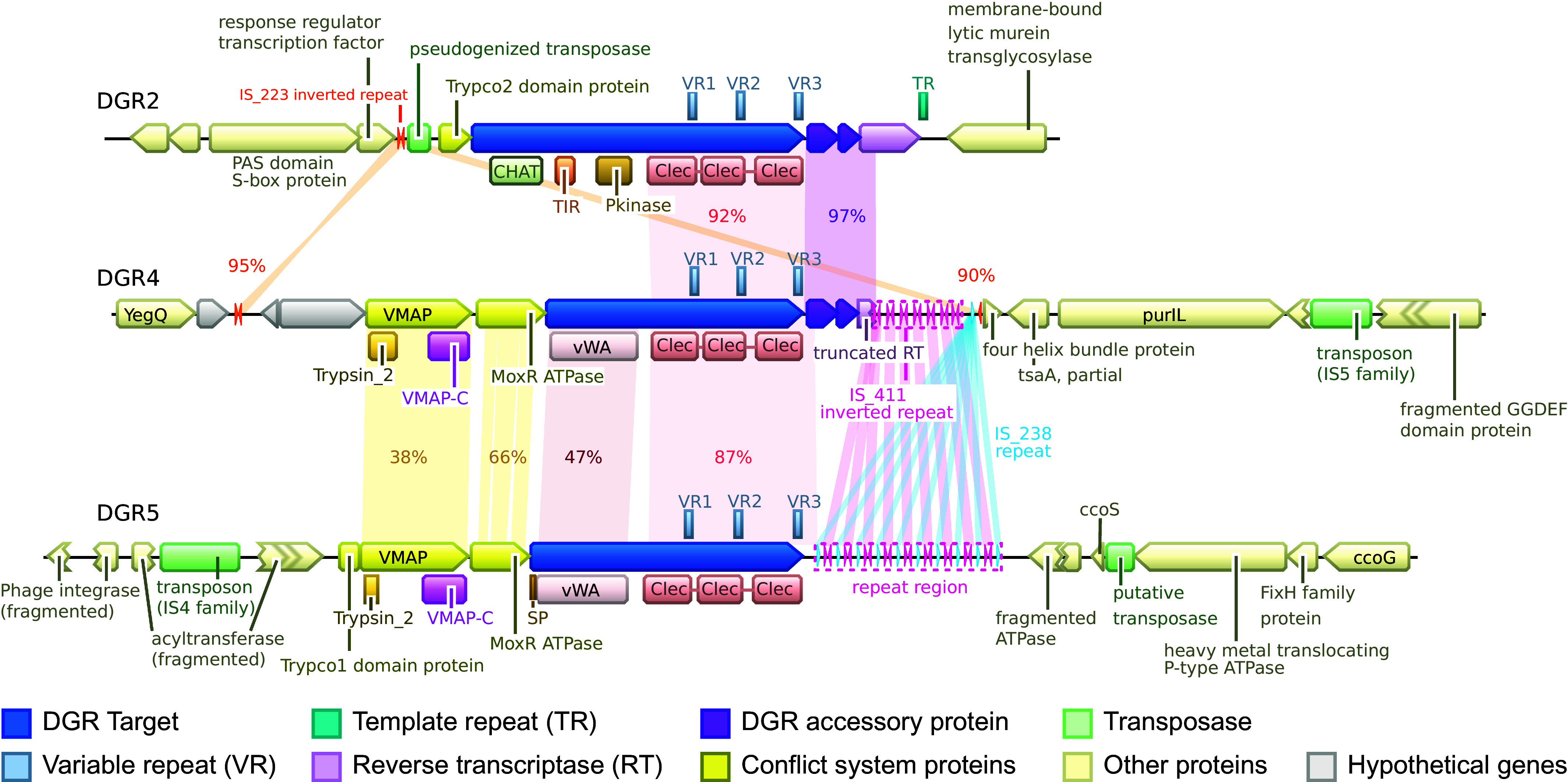
The DGR 2-4-5 loci show signs of recent duplication and transposase activity. The gene neighborhoods surrounding each DGR locus are shown with regions of similarity highlighted along with the percent nucleotide identity. Regions with short direct or inverted repeats (MITE-like) are also highlighted, along with the name of the remote IS elements with matching terminal inverted repeats (IS_223, IS411, and IS_238).

*Thiohalocapsa* PB-PSB1’s DGR loci show the footprints of both duplications and domain shuffling (Fig. 2). The VR-containing, C-terminal CLec domains are quite divergent, but target genes from the same DGR class typically have closely related CLec domains (*SI Appendix*, Fig. S2). In addition, regions of high nucleotide identity, both between and within loci, indicate that recent intragenomic duplications mediated the expansion of the DGR repertoire (Fig. 4 and *SI Appendix*, Fig. S3). Yet, these duplications have not replicated identical DGR loci: the target proteins themselves are diverse and modular. Even within a class, N-terminal regions encode either completely different domains (e.g., DGR 2) or divergent homologs (e.g., the vWA domains of DGRs 4 and 5; Fig. 4 and *SI Appendix*, Fig. S3).

Abundant mobile genetic elements in *Thiohalocapsa* PB-PSB1 (37) appear responsible for the duplication and divergence of DGR loci. The DGR loci contain both complete transposons and pseudogenized transposase genes as well as tandem repeats matching terminal inverted repeats from intact transposons (IS elements) elsewhere in the PB-PSB1 genome (Fig. 4, *SI Appendix*, Fig. S3, and Dataset S4). These tandem inverted repeats resemble miniature inverted repeat transposable elements (MITEs) (Fig. 4, *SI Appendix*, Fig. S3, and Dataset S4). PB-PSB1’s MITE-like sequences are predicted to form stable stem loop RNA secondary structures, a common feature observed in MITEs characterized from bacteria and eukaryotes (50). Arrays of MITE-like repeats have replaced some key functional DGR components, such as the reverse transcriptase gene at DGR 4 and 5 (Fig. 4) or the template repeat of DGR 7 (*SI Appendix*, Fig. S3*C*).

The transposons and MITE-like sequences at these loci are dynamic. Within single pink berry aggregates sequenced deeply with accurate long-read technology (PacBio HiFi), we identified structural variants with intact DGR components alongside variants with transposon-mediated degradation (*SI Appendix*, Fig. S4). At DGR 3, we observed strains that had an intact RT gene coexisting with variants where it was interrupted by a transposon (as in the reference genome assembly). Some strains showed a DGR 7 version where the RT gene and MITE-like array were deleted, and rarer variants contained both the intact RT and a complete TR sequence (*SI Appendix*, Fig. S4).

These examples show the prominent role of mobile elements in the evolution of DGR loci and raise the question of whether these DGRs remain active despite transposase activity and past genomic rearrangements.

### *Thiohalocapsa* PB-PSB1 DGRs Are Diversified in Natural Settings.

To explore whether the PB-PSB1 DGRs have diversified, we examined the sequence variation in metagenomic data from individual pink berry aggregates. We found that the cells of PB-PSB1 within an aggregate have highly similar genomes. Across 184 short-read metagenomes (Illumina) from individual aggregates collected from five sites across three salt marshes (Fig. 1*C* and Dataset S5), average nucleotide identity of reads mapped to PB-PSB1 reference genome ranged from 98.9% to 99.4%. The nucleotide diversity π (the probability for two reads to have a different nucleotide at a given position) within each aggregate was extremely low (median: 0.0022, *SI Appendix*, Fig. S5), with values similar to those observed within a mat of clonally growing *Microcoleus* sp. (51). This corresponded to a median of 2.2 single nucleotide variants per 10 kb. PB-PSB1’s nucleotide diversity was lower for individual aggregates than for the combined population at each site (*SI Appendix*, Fig. S5). These data suggest that binary fission and a viscous biofilm keep PB-PSB1’s clonal kin in close physical proximity.

**Fig. 5.**
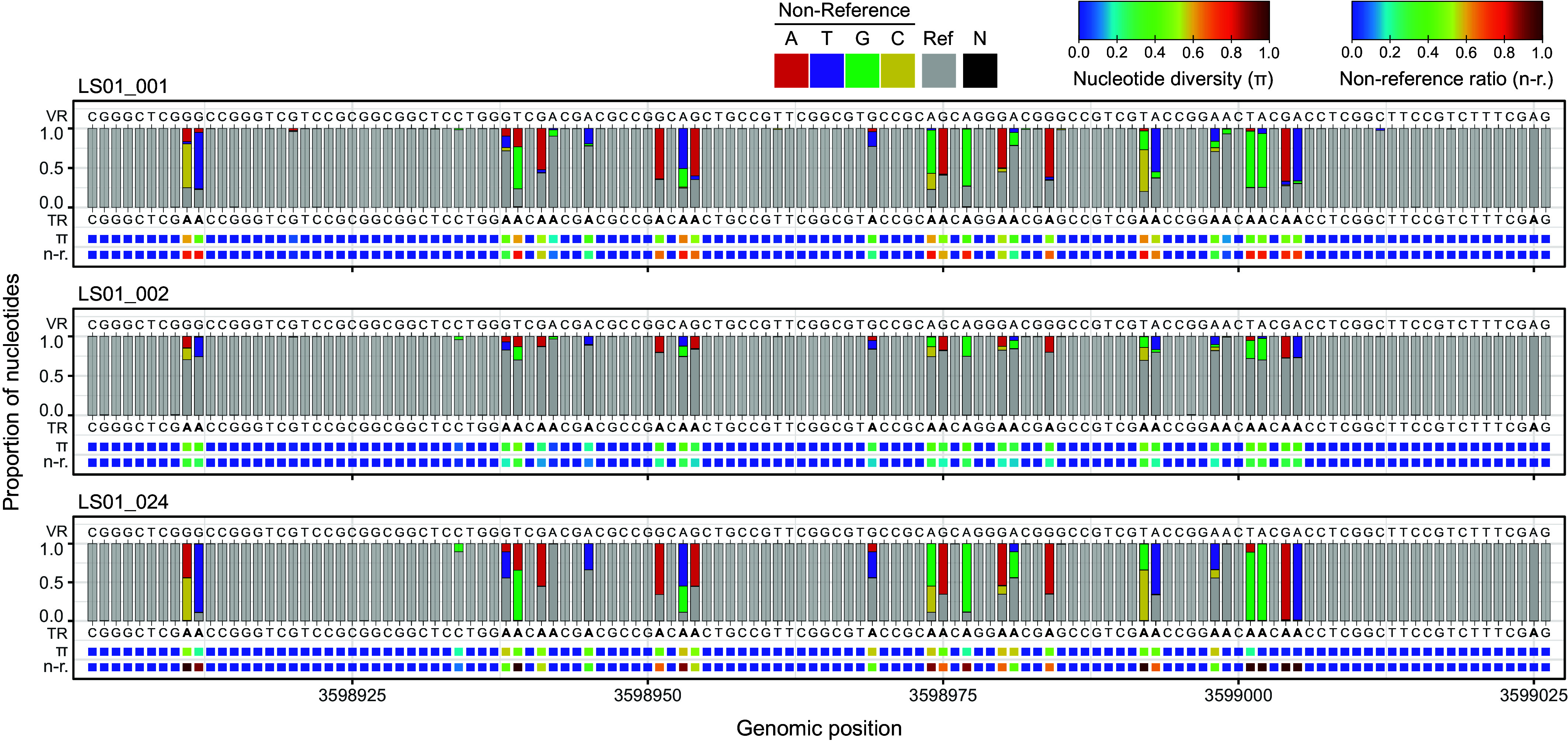
In situ diversification of the VR1 of DGR 4 target in three pink berry aggregates from long-read metagenomic data. Each panel corresponds to an independently sequenced aggregate (LS01_001, LS01_002, LS01_024). Bar plots indicate the proportion of A, T, C, and G nucleotides at each position, colored if they differ from the reference. Letters above bars indicate the VR sequence in the reference genome, while letters below bars indicate the reference sequence of the TR with As highlighted in bold. Bottom rows show the nucleotide diversity (π) and proportion of non-reference alleles (n-r.) at each position. Ref., reference nucleotide; N, unknown nucleotide.

Next, we focused on the sequence variation of the DGR targets. Given the complexity of PB-PSB1’s DGR architecture with multiple duplicated regions, we started by looking for variation in highly accurate, long-read sequencing from three individual aggregates (Fig. 5 and Dataset S5). This analysis revealed within-aggregate variability at the expected target positions of all the DGR loci except for DGR 1. The level of diversification differed between aggregates and across loci, indicating recent diversification (Fig. 5 and *SI Appendix*, Fig. S6). In addition to analyzing nucleotide frequencies, we summarized the variation across DGR repeats using two metrics: i) the proportion of non-reference alleles at a given position and ii) the nucleotide diversity π within an individual aggregate. These metrics allow us to capture diversification at distinct scales: spatiotemporal variation from the reference genome (sampled from location LS01 in 2011) or variation within an individual aggregate (π). High nucleotide diversity of VRs within an individual aggregate could be the sign either of recent DGR activity during the clonal growth of the colony or of the aggregation of individual strains with distinct VR variants.

**Fig. 6.**
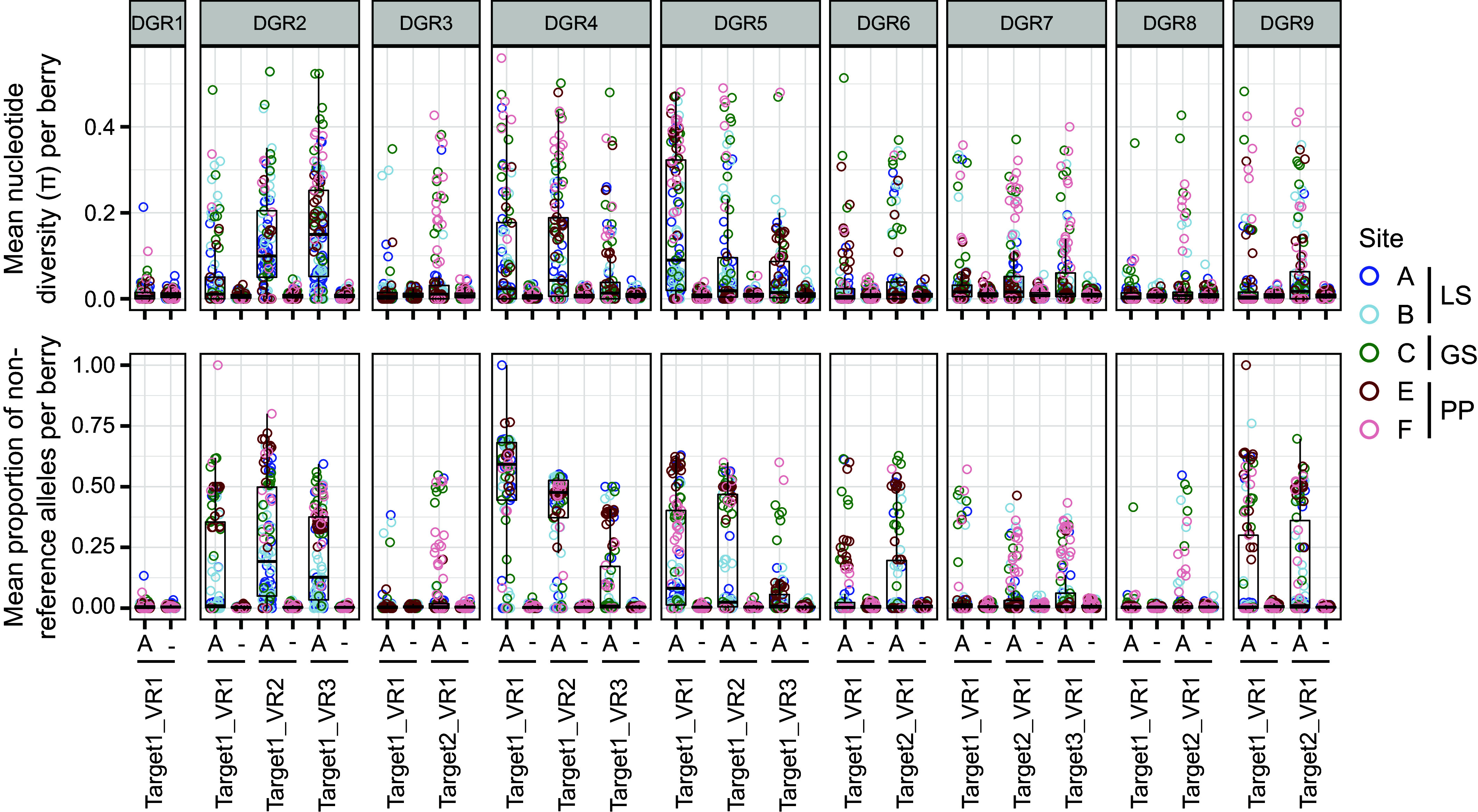
Metagenomics data reveals the differential diversification of PB-PSB1 DGR targets in 184 pink berry aggregates across their geographic range. For each DGR VR, the mean nucleotide diversity (*Upper*) and the mean proportion of non-reference alleles (*Lower*) within an aggregate were calculated separately for positions corresponding to an A in the TR (and thus targeted by mutagenesis, indicated by an A) and for all other positions (indicated by −). Each dot corresponds to a single aggregate colored by the sampling site, blue shades corresponding to Little Sippewissett (LS), green to Great Sippewissett (GS), and red shades to Penzance Point (PP). The boxplots summarize the distribution of values for all aggregates having enough coverage at a given VR.

We then extended our analysis to a higher number of aggregates over the spatial range of pink berries, by searching for DGR variation across our dataset of 184 short-read metagenomes. Consistent with the analysis of long-read data, both the proportion of non-reference alleles and the nucleotide diversity reveal signs of DGR activity at all loci except DGR 1 (Fig. 6). The level of diversity was not correlated with the size of the aggregate (*SI Appendix*, Fig. S7).

In experimentally characterized DGRs, all the system’s components were present at the same genetic locus (*cis* activity) (17, 27). On the contrary, in the PB-PSB1 genome some loci were identified as either incomplete DGRs or candidate remote target genes (DGRs 1, 3, 4, 5, and 7; Fig. 2). As diversification was observed at all but one of these loci (DGR1), we explored the possibility that targets at these loci are diversified in *trans* by an RT and/or TR encoded elsewhere in the genome.

We found some loci where diversification is best explained by *trans* activity, and others where alternate factors could account for the observed diversity. VRs from targets at DGR 4 and 5 perfectly match DGR 2’s TR sequence at all non-adenine sites (*SI Appendix*, Fig. S1*A*), suggesting that these targets are being diversified by DGR 2 components acting in *trans*. However, diversification of the targets of DGR 7 cannot be explained by *trans* activity, as their VRs have no perfect match elsewhere in the genome. As mentioned above, long-read data revealed structural variants at the DGR 7 locus with intact RT and TR components (*SI Appendix*, Fig. S4). At DGR 3, we similarly found a variant with an intact RT gene. We conclude that the diversity we measure at DGR 3 and 7 either occurred prior to the disruption of these loci or represents active diversification in *cis* by the cells possessing intact DGRs.

Despite the lack of variability of the DGR 1 target, its VR matches the DGR 3 TR at all non-adenine sites (*SI Appendix*, Fig. S1*C*). This suggests that DGR 1 may have been able to mobilize DGR 3’s machinery in *trans*. Why diversification has stopped or cannot be observed for DGR 1 remains unclear.

#### Spatial patterns of DGR diversification.

**Fig. 7.**
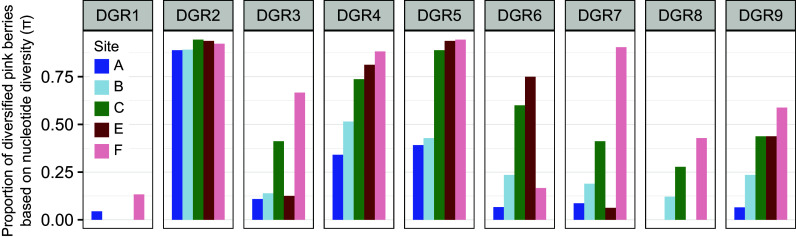
Spatial patterns in DGR diversification. The proportion of aggregates showing diversification is shown for each DGR locus at each sampling site. A DGR locus was considered to be diversified if at least one of its VRs showed diversification at positions targeted by the DGR mechanism based on the nucleotide diversity. Similar results were obtained when using the proportion of non-reference alleles. Colors correspond to sampling sites, with blue shades corresponding to Little Sippewissett (LS), green to Great Sippewissett (GS), and red shades to Penzance Point (PP). *SI Appendix*, Figs. S8 and S9 show these results at the target and VR level as well as the number of diversified aggregates in each case.

We observed spatial patterns in the prevalence of DGR diversification (Fig. 7). While some targets (e.g. DGR 2) were diversified in most aggregates across all geographic sites, others like DGR 3 were diversified frequently at some sites (C, F) but only rarely at others (A, B and E) (Fig. 7). Overall, sites in Little Sippewissett (A and B) showed frequent diversification at only 3 DGR loci (DGR 2, 4 and 5), while diversification was more prevalent at all loci but DGR 1 in other marshes (sites C, E, F).

#### Differential activity of VRs within a DGR locus.

Some PB-PSB1 DGR loci have multiple targets (each with a single VR), while other loci have DGR targets containing multiple VRs. Our metagenomic analysis revealed differences in the level of diversification of distinct targets within a given DGR locus, and of distinct VRs within a given target. At DGR 6, which has two targets, target 1 was diversified less frequently than target 2 at several locations (sites A, B, C and F; *SI Appendix*, Fig. S8). Similarly, at some sites, the third VR in DGR 4’s single target gene was variable more frequently than were the first two VRs (sites A, B and C; *SI Appendix*, Fig. S9). This indicates that even when a corresponding RT and TR are expressed, this does not necessarily lead to equal diversification of all VRs.

Overall, the analysis of this metagenomic dataset revealed that eight of the nine DGR loci in PB-PSB1 are diversified in natural conditions and that the level of diversity of each VR might be dependent on local environmental conditions. The expansion of active DGRs in PB-PSB1 suggests that targeted diversification is highly beneficial to this organism. The spatial variation in activity levels raises the question of the DGR functions and triggers in this organism and in the bacteria with related DGR RTs.

### *Thiohalocapsa* PB-PSB1 and Other Multicellular Bacteria Use DGRs to Diversify Putative Antigen Sensors.

Several of the genes diversified by PB-PSB1’s DGRs have been identified as components of putative biological conflict systems that are enriched in multicellular bacteria (8, 13). We investigated the extent to which all PB-PSB1’s DGRs had this type of association by annotating their neighboring genes. We also explored such associations in the bacteria encoding the DGR loci most closely related to PB-PSB1’s DGRs (shown in Fig. 3).

#### STAND NTPase antiviral defense systems.

PB-PSB1’s DGR 3 and 6 diversify target genes with STAND family NTPase domains, as do many of the other DGRs from clade 5D (Fig. 3). STAND family NTPase domains form the central component of animal and plant innate immune responses as nucleotide-binding oligomerization-like receptors (NLRs). NLRs detect pathogen-associated molecular patterns and trigger programmed cell death via large multiprotein complexes, such as the animal apoptosome and plant resistome (52–54). Recent work demonstrates that bacterial STAND homologs [antiviral STAND (Avs) and bacterial NACHTs] provide protection against ssRNA and lytic and lysogenic dsDNA phages (10, 14). These experimentally characterized antiviral STAND proteins usually show a tripartite domain architecture also found in eukaryotic NLRs, where the STAND domain is surrounded by N-terminal effector domains and a highly variable C-terminal sensor. The C-terminal sensor domains (e.g., tetratricopeptide repeats) bind conserved proteins from tailed phages and trigger cell death *via* their N-terminal effectors (10). Though previously predicted to serve a common purpose (14), CLec domains associated to STAND proteins, like those presented here, have not yet been experimentally characterized.

**Fig. 8.**
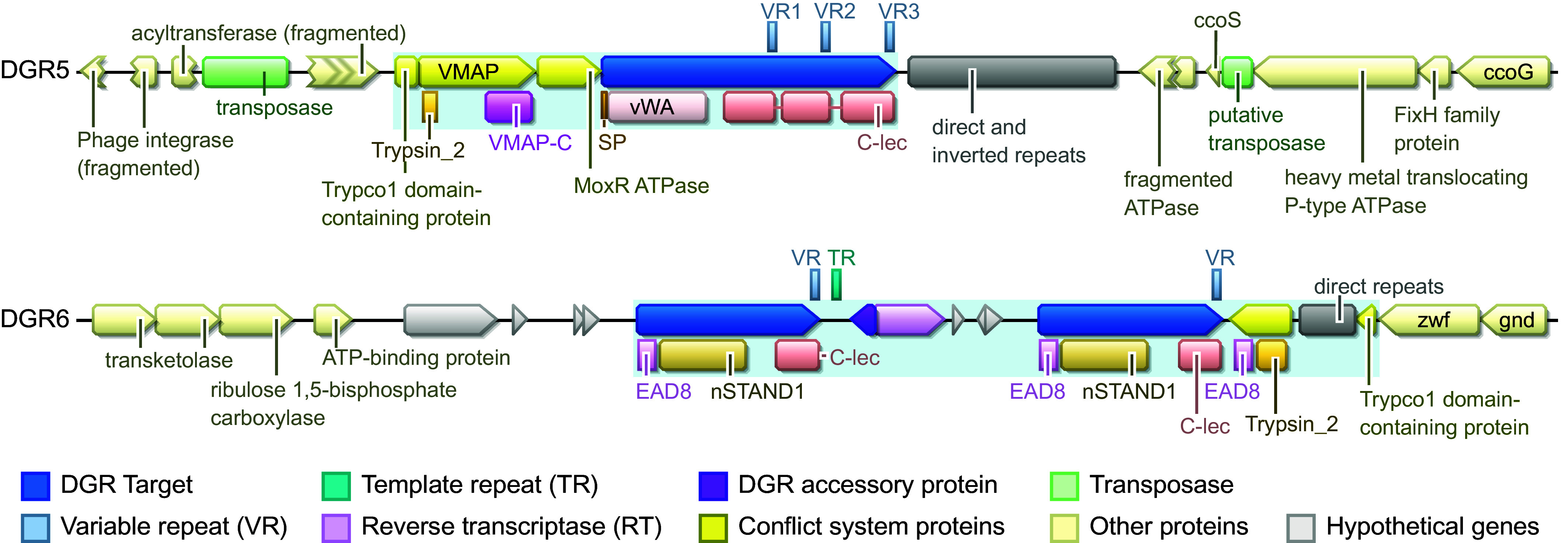
*Thiohalocapsa* PB-PSB1’s DGRs diversify putative antigen sensors in STAND NTPase and ternary conflict systems. Functional annotation of genes surrounding example ternary conflict system type (DGR 5) and STAND NTPase conflict system type (DGR 6) are shown. The light blue shaded areas indicate the putative DGR-containing conflict systems. SP, signal peptide; TM, transmembrane domain. The functional annotation of all DGR loci is reported in *SI Appendix*, Fig. S10.

The DGR 6 architecture was described as a putative conflict system (13), with its two targets containing a STAND NTPase domain (nSTAND1) and an Effector Associated Domain (EAD8), in addition to a C-terminal formylglycine-generating enzyme (FGE) domains, a subtype of CLec folds (Fig. 8). The same EAD8 domain is also found in a nearby protein with a trypsin-like peptidase domain. The CLec domain containing the VR was proposed to be involved in sensing an invasion (13). While characterized antiviral STAND mediate cell death via the nuclease activity of fused N-terminal effector domains (10), here the adapter domain (EAD8) is thought to recruit the trypsin-like protease effector protein by homotypic interactions (8, 13). The DGR 3 locus has a similar domain architecture: duplicated targets each with a central STAND NTPase domain (NACHT) fused to the DGR-diversified CLec domain (*SI Appendix*, Fig. S10). While the targets at the DGR 3 locus do not contain an identified adapter domain, the neighboring S8 family peptidase (N838_12175) has C-terminal tandem WD40 repeats (β-propeller). In Eukaryotes, WD40 β-propellers act as key mediators of protein–protein interactions by serving as scaffolds for multimeric assemblies that mediate diverse functions from cell division to signal transduction and apoptosis (55–57).

DGRs from diverse multicellular bacteria in clade 5D seem to diversify STAND NTPases-containing antigen sensors. Indeed, DGRs in clade 5D commonly had two target genes with single CLec domains and central STAND NTPase domains (nSTAND1 or NACHT), though some used other NTPases types (e.g., KAP NTPase, P-loop NTPases; Fig. 3 and *SI Appendix*, Fig. S11). Many of these targets—like DGR 3—contained N-terminal extensions without previously characterized domains, though some had known N-terminal effectors such as peptidase or TIR domains, which are key components in defense systems and cyclic-oligonucleotide-based antiphage systems (7, 8, 11, 58–60).

#### MoxR-like AAA ATPase ternary conflict systems.

In a recent comparative genomics analysis that predicted novel NTP-dependent conflict systems, *Thiohalocapsa* PB-PSB1’s DGR 7 locus was identified as what the authors term a “VMAP ternary conflict system” (8). This system consists of three central components: a MoxR-like ATPase, a vWA domain protein, and a vWA-MoxR-associated protein (VMAP) (*SI Appendix*, Fig. S10). Kaur et al. proposed that the VMAP proteins, which typically contain either a peptidase, a cyclic nucleotide generating domain, or an adapter domain (EAD), act as sensors of invasion. They predict that the MoxR ATPase transduces this signal by activating the VMAP peptidase, which cleaves the vWA protein to liberate its associated effector domains. We propose an alternate mechanism: the DGR target protein, which contains both a vWA and a variable CLec ligand binding domains, detects an invasion. This sensor then relays the invasion signal through the MoxR ATPase to activate the N-terminal effector of the VMAP protein.

The DGR 7 locus shows the three central components, with an N-terminal trypsin-like peptidase effector domain on the VMAP protein. The DGR-diversified putative sensor has both a vWA and a VR-containing CLec domain, with additional signal transduction domains (a 3′,5′-cyclic AMP phosphodiesterase domain and a NACHT domain, *SI Appendix*, Fig. S10). The DGRs of the few cultured representatives in clade 5C did not resemble ternary conflict systems (Fig. 3). However, the DGR loci most closely related to DGR 7 from metagenomic sources (including PB-PSB1’s DGR 9) have characteristic components of ternary systems. Unbinned metagenomic contigs (21) found in aquatic and wastewater treatment habitats had both a MoxR ATPase and N-terminal vWA domains in the target genes (*SI Appendix*, Fig. S11). DGR 9 has a related architecture: a DGR target gene directly adjacent to a MoxR ATPase and a trypsin-like peptidase. Here, though, the putative sensor has an unknown N-terminal extension in place of the vWA domain, and the canonical VMAP domain was not found in the C-terminal region of the peptidase (*SI Appendix*, Fig. S10).

The DGR 4 and DGR 5 loci have a single target gene and display the typical architecture of the VMAP ternary system. At these loci, the target protein has an N-terminal vWA domain followed by three tandem CLec domains. Among the closest relatives of DGR 4 in the RT phylogenetic tree (clade 5B, Fig. 3), only *Marichromatium purpuratum* 984 and an unbinned *Chromatiales* contig possess a similar architecture of a single target gene with tandem VR-containing CLec domains that are part of a VMAP system. These target genes are syntenic with distinct vWA-domain containing genes, a MoxR-type ATPase, adapter domains (e.g., Trypco1) and effector domains (e.g., trypsin-like peptidase, TIR), as seen at the DGR 4 and 5 loci (Fig. 8 and *SI Appendix*, Figs. S10 and S11).

In the other loci of clade 5B, including PB-PSB1 DGR 2, there is no identified association of the DGR with a complete VMAP system. The DGR 2 locus contains an adapter domain (Trypco2; *SI Appendix*, Fig. S10) that has been described in conflict systems that use trypsin-like peptidase effectors (8). The DGR 2 target itself contains a caspase-like CHAT proteolytic domain and a TIR domain, which are key effector domains in bacterial defense systems (7, 8, 11, 58–60).

The DGR 8 locus represents a variation on this “ternary” system that is commonly associated with the large clade 5A of RTs (Fig. 3). DGR 8 and other members of clade 5A have a MoxR-like ATPase immediately upstream from a target gene but in place of a vWA-domain, their target genes have N-terminal extensions without predicted domains (*SI Appendix*, Fig. S11). DGR 8 and its closest relative *T. drewsii* have a helix-turn-helix domain gene in place of the classic ternary system VMAP, suggesting an alternate effector mechanism (*SI Appendix*, Fig. S11). Most of the other loci in this clade 5A had a similar organization to *Thiohalocapsa*’s DGR 8, including two targets with C-terminal CLec domains: one short CLec-only target and another with a long N-terminal extension (*SI Appendix*, Fig. S11). Clade 5A loci also often had adjacent genes with adapter (Trypco1, Trypco2) and effector (TIR, trypsin-like or caspase-like peptidase) domains (Fig. 3).

Most of the DGRs from multicellular cyanobacteria in clade 5E also seem to diversify putative sensors for VMAP ternary systems. These DGRs are commonly associated with a MoxR ATPase, effector domains (peptidases, TIR), adapter domains, and vWA domain-containing targets, though only a subset possesses a conserved VMAP domain (Fig. 3). Cyanobacterial DGRs of this clade were previously speculated to have a role in signal transduction and cell death (31). We propose that the signals detected are antigens and that the DGR-diversified targets are the sensors.

#### Predicted cellular localization.

In the PB-PSB1 genome, the DGR target proteins are predicted to have different cellular localizations, which, if they act as sensors as we predict, would make the cell poised to detect threats in both the cytoplasm and periplasm (Fig. 8 and *SI Appendix*, Fig. S10). Among the MoxR ATPase-associated DGRs, the targets of DGR 2, 4, 7, and 8 are predicted to be cytoplasmic, while DGR 5’s target is likely secreted or on the external surface, as it has a signal peptide but no transmembrane domain. DGR 9’s targets contain multiple transmembrane regions that would position the CLec domains in either the cytoplasm (target 2) or the periplasm (target 1). Similarly, in PB-PSB1’s STAND NTPase systems, the DGR 1 and 6 targets are likely cytoplasmic, while the NACHT and CLec domains of the DGR 3 locus are predicted to be cytoplasmic in the first target and periplasmic in the second target.

## Discussion

### The Exceptional Diversification Potential of PB-PSB1.

The multicellular bacterium *Thiohalocapsa* PB-PSB1 has an unusual capacity for diversification. Using DGRs, PB-PSB1 can generate 10^282^ different protein combinations by mutating specific positions within fourteen DGR targets that we found were diversified in the natural environment. DGR systems are rare, found in only 2% of complete genomes from public databases, and have a sporadic distribution across diverse bacterial and archaeal phyla (21, 30). Organisms with multiple DGR systems are even rarer: among organisms with DGRs, only 2% have more than two complete systems (21). Despite the overall rarity of DGRs across the tree of life, we found that multicellular bacteria like PB-PSB1 often have two or more DGRs from a single monophyletic clade (*SI Appendix*, Table S1). PB-PSB1 and other multicellular bacteria with multiple clade 5 DGRs are exceptional in that they contain not just numerous targets but several complete, independent DGR systems.

Prior work identified a similar expansion of DGR loci, often with multiple independent loci in a genome, among the archaea from the DPANN superphyla and the bacteria of the Candidate Phyla Radiation (CPR) (20). In CPR bacteria, the most commonly observed architecture of DGR target domains was a CLec domain fused to an AAA ATPase (20), potentially indicating a similar molecular mode of action where the ATPase domain acts as a transducer for signals detected in the C-terminal region. However, most of the RT sequences from CPR bacteria form an independent monophyletic clade distant from clade 5 (RT clade 2 in ref. 21). The RTs from CPR bacteria and DPANN archaea found in clade 5F (Fig. 3) are phylogenetically closer to RT clade 6, composed of viral DGR RTs (21). As these clade 5F CPR/DPANN DGRs are not associated with conflict system domains (Fig. 3), they likely play a different cellular role than other clade 5 DGRs [e.g., mediating their interactions with the host cell (20)]. In contrast, we demonstrate here that a specific lineage of DGRs (clades 5A-5E) can be recruited by diverse taxa to diversify the putative sensor proteins of programmed cell death conflict systems (8, 10, 13, 14).

### Diversification Is Tightly Constrained by the Local Environment.

Comparing PB-PSB1 from different marsh pools, we observed spatial patterns in the level of diversity among its different DGR target genes. A prior analysis of metagenomic time series data showed that most DGRs were used to maintain a relatively constant level diversity in the target genes (21). While diversity may be stable through time, we find that the level of diversity is highly site-dependent. Indeed, the level of population diversity for each DGR target (and even of each VR within a target gene) depends on the location of the pink berry aggregates in salt marshes less than 10 km apart. The diversity we measure at these loci is a function of both diversification (via DGR mutagenesis) and selection of specific variants. The spatial patterns we observe are thus likely driven by a combination of differential activity of DGR regions depending on local environmental conditions and differences in the selection pressures at each site.

At some sampling sites, select DGR targets showed very low levels of diversity (e.g*.,* DGR 3 and 8 in sites A, B and LS01, Fig. 7 and *SI Appendix*, Fig. S6), which could indicate either a recent local selective sweep or DGR inactivity due to repression or disruption of these systems. With deep long-read sequencing from additional sites to phase the single nucleotide variants across a VR, these processes could be differentiated by examining whether distinct alleles are present at different geographic sites. At a much larger spatial scale, populations of the multicellular cyanobacterium *Trichodesmium erythraem* in the Indian and Pacific oceans were shown to harbor distinct combinations of DGR target gene alleles, suggesting differential selection (61).

What ecological factors might alter the selection pressures on PB-PSB1 DGR targets? Recent work revealed that viral communities can change at the sub-kilometer scale in interconnected coastal wetlands (62). In a study describing pink berry phages, two dsDNA lytic phages infecting *Thiohalocapsa* PB-PSB1 were found to have varying relative abundance between individual aggregates from the same pond (63). PB-PSB1 was also shown to have encountered numerous unknown phages, indicating that infection is likely a meaningful (if still largely uncharacterized) threat (63). If PB-PSB1’s DGR targets are used in defense systems, as we propose, viral spatiotemporal dynamics could play a critical role in shaping the adaptive landscape for these loci.

Of note, our results showed some population heterogeneity in the disruption of DGR loci, demonstrating that they may experience a balance between relaxed selection (allowing for the degradation of the system), and selection for functional, diversifying systems. Regardless of the underlying mechanism, the spatial pattern of diversification highlights the importance of exploring DGR activity in various environmental or experimental conditions (21, 61).

### The Role of Transposons in the Evolution of DGRs.

The phylogeny of DGR RTs suggests that PB-PSB1 acquired DGR systems separately from distant organisms (Fig. 3). Horizontal transfer of DGRs between phylogenetically distant bacteria has been repeatedly observed and prior studies highlighted the role of phages, plasmids, and transposons as putative vectors (18, 20, 25, 29, 31, 64, 65). Transposons have been linked to the mobilization and horizontal transfer of a DGR system between *Gammaproteobacteria* (64) and were commonly found alongside DGRs in numerous CPR bacteria and DPANN archaea (20). While transposons may enhance the dispersal of DGRs, they are not without risk: transposons and MITE-like elements degraded redundant components of PB-PSB1’s DGR loci and were found to truncate DGR RT genes in a prior study (64).

How do new DGR target genes evolve? Duplication and recombination has been proposed as a mechanism to generate novel target proteins by adding VR-containing CLec domains to the C-terminus of diverse proteins (21, 27, 31, 64). In the PB-PSB1 genome, several DGR loci seem to have diversified through intragenomic duplication and recombination (Fig. 4 and *SI Appendix*, Fig. S3). Transposons could be responsible for these duplications either by translocating parts of DGRs as a cargo or by triggering homologous recombination between their numerous copies distributed within the genome. The example of DGR 2-4-5 is particularly eloquent: the RT, accessory genes, and the target gene C-terminal region have been duplicated and recombined with distinct N-terminal domains, while MITE-like tandem repeats have degraded redundant DGR components (Fig. 4). PB-PSB1 provides a striking example of how the evolution of DGRs combines two distinct dimensions of variation commonly seen in adaptive immune systems: domain shuffling and hypermutation.

### DGR Targets as Sensors in Conflict Systems of Multicellular Bacteria.

Our manual annotation of DGR targets and surrounding genes revealed that most, if not all, of the PB-PSB1 DGRs are parts of putative conflict systems where we believe the diversified CLec domains of the DGR targets are used as antigen sensors. Within each conflict system, it is likely that the proteins associate into a complex (8, 10, 13), thus increasing the avidity of the CLec domains toward specific antigens (66, 67). Among experimentally described antiviral STAND NTPase systems, binding of viral-associated molecular patterns initiates complex formation and programmed cell death (10), an effector response conserved across animal and plant innate immune responses and also proposed for ternary conflict systems (8, 13). In PB-PSB1, the DGR-diversified domains have different predicted cellular localizations that would equip bacteria to sense invasions in all cellular compartments. This proposed interaction between DGR targets and invading antigens raises the intriguing question of how these diversified sensors distinguish between self vs. non-self antigens to avoid autoimmunity.

Such anticipatory ligand binding mediated by sensors undergoing targeted mutation is rare and has been experimentally demonstrated only in the vertebrate adaptive immune system and in the viral receptor-binding protein where DGRs were discovered (17, 18, 68). However, we propose that this may be a common purpose for one of the major cellular lineages of DGRs (clade 5). We found that a striking proportion of DGRs from clade 5 are encoded by diverse bacteria with multicellular or aggregative lifestyles (Fig. 3 and Dataset S3). These bacteria often had multiple DGR systems that diversify similar putative antigen sensors with CLec domains associated with NLR domains, adapter domains and adjacent effector proteins (Fig. 3). In an arms-race scenario, the benefits of hypervariable antigen sensors for the surveillance and response to novel threats are clear, particularly to multicellular bacteria: high cell density and limited genetic diversity makes them especially vulnerable to attacks by viruses or mobile DNA/RNA. The potential parallels to the vertebrate adaptive immunity are striking: 1) diversification of a pattern recognition sensor from innate immunity, 2) targeted mutation restricted to a short region of the sensor gene via error-prone DNA synthesis/repair, and 3) clonal expansion of cells with high-affinity sensors (34, 69).

These parallels raise interesting evolutionary questions about the evolution of multicellularity. We propose that infection represents a universal evolutionary constraint for all cellular aggregations of close genetic relatives. The risk of infectious epidemics in an aggregate with little genetic diversity creates a strong selective pressure for successful multicellular life forms, from bacteria to metazoans, to evolve sophisticated immune responses that include threat surveillance by highly diversified sensors, as well as programmed cell death. In eukaryotes, programmed cell death is seen as necessary for multicellular life (52), and in model simulations, the emergence of programmed cell death was tied to the evolution of multicellularity (70).

Did these systems first emerge in a unicellular ancestor or were they evolved as an explicit adaptation to multicellularity? Nedelcu et al. provide a framework for the puzzling conundrum of programmed cell death in unicellular organisms (71). The genes triggering cell death under certain conditions could, in fact, be maladaptive but persist as a byproduct of selection for their normally beneficial housekeeping functions. While premature death may have been an unfortunate side effect for unicellular ancestors, this function could be co-opted and refined as an altruistic defense under conditions favoring kin/group selection. The pink berry’s viscous biofilm and low genetic diversity create such conditions, where the death of an infected cell would directly benefit adjacent (nearly) clonal siblings.

In addition to a vulnerability to attacks, multicellularity means sharing resources with neighbors, introducing a selection pressure for kin recognition (72). In fungi where cell fusion and syncytial organization are common, some species use NLR homologs to prevent fusion of genetically dissimilar strains by initiating programmed cell death, a process known as heterokaryon incompatibility (10, 14, 73). These polymorphic hetero-incompatibility determinants sense proteins from dissimilar strains using their highly variable C-terminal ligand binding domain (TPR or WD-repeats) and transduce this signal via a central STAND ATPase domain, triggering oligomerization and activation of N-terminal effectors (72, 73). These similarities to the systems presented here suggest an intriguing alternative role for the DGR-diversified conflict systems in multicellular bacteria: to permit the association with kin and avoid associations with non-kin cells, in a similar fashion to fungi hetero-incompatibility or bacterial kin recognition systems (72, 74).

## Conclusions

Here, we used *Thiohalocapsa* PB-PSB1 to study the association of DGRs with bacterial conflict systems. This organism has an unusual abundance of DGRs, bringing a staggering potential for targeted diversification. PB-PSB1’s DGR reverse transcriptases belong to a large monophyletic clade of DGRs from diverse multicellular bacteria. We propose that these DGR targets act as variable antigen sensors in conflict systems triggering programmed cell death, either as a defense mechanism or for kin recognition. We show that 14 target proteins of PB-PSB1 DGRs are diversified to differing degrees across the species’ known geographic range, with spatial patterns likely driven by the combined effects of DGRs’ regulation and differential selection. Our work suggests that hypermutation of sensors for anticipatory ligand binding may represent an important adaptation to the constraints of multicellular life and calls for experimental characterization of these systems in multicellular bacteria.

## Material and Methods

Extended methods are available in *SI Appendix*, *Supporting Text*. A total of 187 pink berry aggregates were sampled from 6 ponds across 3 salt marshes near Woods Hole, MA (Fig. 1 and Dataset S5). A total of 184 aggregates sampled between 2015 and 2017 were sequenced on an Illumina HiSeq 2500 (250 bp paired-end reads) at the Whitehead Institute for Biomedical Research (Cambridge, MA). Illumina reads were cleaned with bbduk.sh in bbmap v38.92 (https://sourceforge.net/projects/bbmap/) with options forcetrimright2=30 qtrim=rl trimq=20 maq=20 minlen=50. Three additional aggregates sampled in 2021 were sequenced for long-reads metagenomics on a Pacific Biosystem Sequel IIe sequencer. CCS reads were filtered for duplicates using pbmarkdup, and BBMap was used to remove potential chimeric reads, and trim adapters.

DGRs were detected in the *Thiohalocapsa* PB-PSB1 genome (GenBank GCA_016745215.1) using MyDGR with default options (75) and as described in ref. 20 using the package at https://pypi.org/project/DGR-package/ (20, 21). Loci were manually inspected and refined. A custom python script (https://doi.org/10.5281/zenodo.10569842) (76) was developed to calculate all possible protein sequence combinations for each VR of each DGR. Insertion sequence (IS) elements in *Thiohalocapsa* PB-PSB1’s genome were annotated using ISEScan v1.7.1 with default parameters (77) (Dataset S4). MITE-like sequences were identified by manual inspection of dotplots and predicted secondary structure.

The closest relatives of PB-PSB1’s DGRs were identified as members of RT clade 5 [*sensu* (21)] by comparison to a published reference database of 1143 representative RT sequences using the phylogenetic workflow previously described (21). We built a phylogeny from a refined set of clade 5 RT (Dataset S3) by removing metagenomic or single-cell genomes (except for those present in the IMG Genomes database). These 257 amino acid sequences were aligned with MAFFT v7.407 (78) in einsi mode and trimmed using TrimAl v1.4.rev15 (-gappyout) (79). The tree was inferred with IQ-Tree v1.5.5 (38) using the built-in model selection (optimal model: Q.pfam+F+R10) (80) and 1000 bootstrap replicates. iTOL (81) was used to visualize select conflict system-associated domains within 20 kb of the DGR RT that were identified either from the IMG annotations of PFAM domains or via *hmmscan* (82) (Dataset S6).

Gene neighborhoods surrounding *Thiohalocapsa* PB-PSB1’s DGR loci and those from a selection of genomes and metagenomic contigs from clades 5A-5D were manually inspected to refine their annotations. Sequences were run through InterProScan (83), hmmscan in the HMMER Web server (84) and NCBI Conserved Domains Database (85, 86) to predict functional domains. Protein sequences were compared to the domains described in refs. 8 and 13 using *hmmscan* in HMMER 3.3.2 (82), based on PFAM HMM profiles and the vWA-ternary domain alignment (Dataset S7).

To detect the footprints of DGR activity, the PacBio HiFi and Illumina reads were mapped to the PB-PSB1 genome using minimap2 v2.24-r1122 (option -ax map-hifi) (87) and *bwa mem* v0.7.17-r1188 (default options) (88), respectively. Mapped reads were extracted with samtools (89). To prevent nonspecific mapping between similar DGR targets, a custom python script was used to extract only those Illumina reads with a partial match within a DGR VR whose mate mapped within 1 kb. The nucleotide frequencies across the genome were computed using *samtools* *mpileup* (option -a). Custom python scripts were used to extract nucleotide frequencies from each VR position with >5× coverage and to calculate the proportion of non-reference alleles and the nucleotide diversity [π = 1-(a^2^+t^2^+c^2^+g^2^) (90) at each position. We considered a VR to be diversified in a given “pink berry” aggregate’s metagenome if the $mean\left(\pi_{A}\right)>mean\left(\pi_{\mathrm{nonA}}\right)+2*sd\left(\pi_{\mathrm{nonA}}\right)$ , where $\pi_{A}$ is the mean value of nucleotide diversity at VR positions targeted by DGR and $\pi_{\mathrm{nonA}}$ the mean value of nucleotide diversity at VR positions *not* targeted by DGR. The same formula was applied for the proportion of non-reference alleles. When aggregating results at the target- or DGR-level, a locus was considered as diversified in an aggregate if at least one of the VRs of this locus showed variability. Alignment of PacBio long reads were used to detect structural variants at DGR loci by manual inspection using IGV (91) and GenomeRibbon (92). In the case of DGR 7, a read representing the structural variant with an intact TR was identified in this manner and annotated using MyDGR (*SI Appendix*, Fig. S4*D* and Dataset S7).

## Supplementary Material

Appendix 01 (PDF)

Dataset S01 (XLSX)

Dataset S02 (XLSX)

Dataset S03 (XLSX)

Dataset S04 (XLSX)

Dataset S05 (XLSX)

Dataset S06 (XLSX)

Dataset S07 (GZ)

Dataset S08 (GZ)

## Data Availability

All new sequencing data are available in NCBI GenBank under BioProject PRJNA1019683 (93) and JGI’s IMG database (3300056627, 3300056928, 3300056818) (94–96). Accession numbers are provided in Dataset S5. Code has been deposited in GitHub (https://doi.org/10.5281/zenodo.10569842) (76).
