# Supplementary material for: Targeted hypermutation of putative antigen sensors in multicellular bacteria
Source: Proc Natl Acad Sci U S A. 2024 Feb 14;121(9):e2316469121. doi: 10.1073/pnas.2316469121 (PMC10907252; doi:10.1073/pnas.2316469121)
Supplement: Supplementary file 1 — Appendix 01 (PDF) [file pnas.2316469121.sapp.pdf]

Supporting Information for

Targeted hypermutation of putative antigen sensors in multicellular bacteria

H. Doré, A. R. Eisenberg, E. N. Junkins, G. E. Leventhal, Anakha Ganesh, O. X. Cordero, B. G. Paul, D. Valentine, M. A. O'Malley, E. G. Wilbanks*

*Corresponding author: Prof. Elizabeth G. Wilbanks
Email: ewilbanks@ucsb.edu

This PDF file includes:

Supporting text
Legends for Datasets S1 to S8
Table S1
Figures S1 to S11
SI References

Other supporting materials for this manuscript include the following:

Datasets S1 to S8

Supporting Information Text

Extended Results

Co-evolution between target genes and their cognate TR
As was noted in previous studies, we observed that the positioning of the adenines in the TR is tightly constrained both to maximize diversification and avoid stop codons in the target protein (SI Appendix Fig. S1, SI Dataset S2) (1–4). This highlights the strong coevolution between the target genes and their cognate TR (5). Indeed, to ensure that the DGR target gene stays functional while being diversified, selection must act on TR sequences to maintain several features: 1) An A at 1st and 2nd positions of codons that can be diversified without compromising the target protein integrity, 2) non-A and non-G nucleotides at the 3rd position of targeted codons to avoid nonsense codons, and 3) non-As for codons that are essential and cannot be diversified. Arguably, these features could be used to recognize DGR systems under active selection.

The fact that the mutation process in DGR is not fully random but directed to the first and second positions of target codons to maximize protein diversity also explains the enrichment in non-synonymous variants in VRs reported from metagenomics analyses (6). As a consequence of this non-randomness, classical measures of selection based on dN/dS or pN/pS cannot be used to estimate the strength of the selection affecting DGR targets.

Diversity of organisms with clade 5 DGRs
Clade 5D, which includes RTs from *Thiohalocapsa* PB-PSB1's DGR3 and DGR6 loci, was composed of entirely of multicellular bacteria and a large proportion of the organisms with multiple DGR loci (SI Appendix Table S1 and SI Dataset S3). Basal members of this clade, most closely related to DGR3, are filamentous chloroflexi (7–9). Derived members of this clade, more closely related to DGR6, are predominantly *Betaproteobacteria* including numerous *Accumulibacter* species from freshwater wastewater treatment reactors and marine *Nitrosomonas* that form dense microcolonies in biofiltration biofilms (10–12). Other members include the mat-forming purple sulfur bacterium *Thioflavicoccus mobilis* and a multicellular magnetotactic bacterial species from the *Deltaproteobacteria* (*Candidatus* Magnetomorum HK-1) (13, 14).

In several cases, we found organisms encoding both clade 5D and clade 5A RTs, like *Thiohalocapsa* PB-PSB1 (Fig. 3, SI Appendix Table S1). 77% of the Clade 5A sequences from described species were from multicellular organisms. This clade, like 5D, includes mostly beta- and gammaproteobacterial species from the *Accumulibacter*, *Nitrosomonas* and purple sulfur bacteria. PB-PSB1's clade 5A RT (DGR8) is most closely related to the mat-forming *Thiorhodococcus drewsii*, which was isolated from the neighboring Great Sippewissett salt marsh (15) and the purple sulfur bacterium *Ca.* Thiodicyton syntrophicum, which, like PB-PSB1, forms multicellular consortia with a sulfate reducing symbiont (16).

In Clade 5B, the relatives of the DGR2 and DGR4 RTs are found in the genomes of known or putative sulfur oxidizing bacteria and, of those that have been visually characterized, all are either filamentous or aggregate forming (Fig. 3, (17–22)). The closest cultured relative to the DGR 2-4 RTs is the purple sulfur bacterium *Marichromatium purpuratum* (also from the family *Chromatiales*). Other close relatives from metagenomic data include a 40 kb *Chromatiales*-like contig from a meromictic lake (Lake La Cruz, Spain).

PB-PSB1's DGR 7 and 9 RTs belonged to clade 5C and had few close relatives amongst cultured or high-quality MAGs in the IMG genomes database, except for *Ca*. Accumulibacter phosphatis BA-91, which also encodes a type 5D DGR (Fig. 3). Close RT relatives from unbinned metagenomic contigs (6) came from other aquatic and wastewater treatment habitats. Unlike PB-PSB1 and *Ca*. Accumulibacter phosphatis BA-91, the DGRs from the unicellular members of clade 5C were located within predicted prophage regions and targeted genes without CLec domains (or, often, any known domains, Fig. 3).

Extended Methods

DNA extraction and sequencing

To analyze the diversity of PB-PSB1 in its natural environment, 187 pink berry aggregates were sampled from 6 ponds across 3 salt marshes near Woods Hole, MA (Figure 1, Supplemental Data 5). 184 metagenomes from individual aggregates sampled between 2015 and 2017 were sequenced with short-read sequencing technology. DNA was extracted from all samples with the Agencourt DNAdvance Genomic DNA Isolation Kit (Beckman Coulter, Indianapolis, USA). Metagenomic libraries were prepared with the Nextera XT DNA Library Prep Kit and Illumina index primers (Illumina, San Diego, USA). Libraries were quantified on an Agilent 4200 TapeStation system with High Sensitivity D5000 ScreenTapes (Agilent Technologies, Santa Clara, USA) and pooled by equimolar amounts. Sequencing was performed on an Illumina HiSeq 2500 machine (250bp paired-end reads) at the Whitehead Institute for Biomedical Research (Cambridge, MA). The resulting Illumina reads were cleaned with bbduk.sh in bbmap v38.92 (https://sourceforge.net/projects/bbmap/) in two steps. First, the last 30bp at the 5' end of reads were removed with option forcetrimright2=30. Then read ends were trimmed based on quality (Q20) and only reads longer than 50 bp with an average quality above Q20 were kept (options qtrim=rl trimq=20 maq=20 minlen=50).

Three additional aggregates sampled in 2021 were sequenced with PacBio HiFi long-read technology. After sampling, aggregates were rinsed with 0.22 μm-filtered seawater and frozen at -80 °C until DNA extraction. In order to maintain DNA integrity for long-read sequencing, a DNA extraction protocol was adapted from (23) and (24). Briefly, a single frozen aggregate was ground in a 1.5 mL tube and incubated at 37 ºC for 1h with 125 μL of Tris Lysis Buffer with extra EDTA (100 mM NaCl, 10 mM Tris HCl at pH 8, 100 mM EDTA at pH 8, 0.5% w/v SDS) and 10 μL of lysozyme at 100 mg/mL. After addition of 125 μL of warm 4% high-salt CTAB (4% w/v CTAB, 10 mM Tris HCl pH 8, 100 mM EDTA pH 8, 2.8 M NaCl), 6 μL of proteinase K (20 mg/mL, New England Biolabs, Ipswich, MA, USA) and 0.8 μL RNAse A (New England Biolabs, Ipswich, MA, USA), the tube was incubated at 55 ºC for 3 hours. After cooling down, 250 μL of chloroform:isoamyl alcohol (24:1) were added before mixing for 15 min on a rotator mixed. The aqueous and organic phases were then separated by spinning for 15 min at 6000 g, and the aqueous phase was carefully transferred to a fresh 1.5 mL tube. This cleaning step was repeated a second time to remove any trace of proteins. Then, 450 μL of warm CTAB precipitation buffer (2% w/v CTAB, 50 mM Tris HCl pH 8, 100 mM EDTA pH 8) were added. After overnight incubation at 55 ºC, the precipitated DNA was harvested by centrifugation at 16000g for 15 min. The DNA pellet was rinsed twice with cold 80% ethanol before elution in 10 mM Tris HCl, pH 8.

To prepare PacBio HiFi libraries, an input of 50 ng of genomic DNA was sheared to 6 kb - 10 kb using the Megaruptor 3 (Diagenode). The sheared DNA was treated with an exonuclease to remove single-stranded ends, a DNA damage repair enzyme mix, and an end-repair/A-tailing mix, and then ligated with amplification adapters using SMRTbell Express Template Prep Kit 2.0 (PacBio). Templates were purified with ProNex Size-Selective Purification System (Promega). The purified ligation product was split into two reactions and enriched using 10 cycles of PCR using the SMRTbell gDNA Sample Amplification Kit (PacBio). The amplified product was combined and treated with a DNA damage repair enzyme mix and an end-repair/A-tailing mix and ligated with barcoded overhang adapters. Libraries were size-selected using the 0.75% agarose gel cassettes with Marker S1 and High Pass protocol on the BluePippin (Sage Science). The PacBio Sequencing primer was then annealed to the SMRTbell template library and sequencing polymerase was bound to them using Sequel II Binding kit 2.0. The prepared SMRTbell template libraries were sequenced on a Pacific Biosystem Sequel IIe sequencer using SMRT Link 10.2, tbd-sample dependent sequencing primer, 8M v1 SMRT cells, and Version 2.0 sequencing chemistry with 1x1800 sequencing movie run times. CCS reads were processed with the JGI QC pipeline to remove artifacts. Briefly, reads were filtered for duplicates using pbmarkdup, analyzed using the icecreamfinder.sh script in BBMap to filter potential chimeric reads, and adapter trimmed using bbduk.

Details on the annotation of PB-PSB1 DGR loci

Manual inspection of DGR loci revealed that, while myDGR correctly predicted the DGR7 locus based on the presence of the RT gene, the target genes and VR/TR regions were misidentified. As DGR7 and 9 had closely related RT sequences, we aligned the CLec domains from their adjacent genes with the predicted TR from DGR9 to identify the target genes and VR at DGR7. ISEScan (25) predicted an IS elements within each of these three target genes of the DGR7 locus; however, a careful inspection revealed that these IS elements contained a lone 3',5'-cyclic AMP phosphodiesterase domain rather than a transposase domain and had poorly matched or absent terminal inverted repeats. They represented an overprediction by ISEscan, caused by the repetitive nature of the region and their proximity to a

downstream transposase fragment, and were removed from the annotation presented in supplemental figures S3 and S8.

Calculation of the number of possible protein sequence combinations

The custom python script (available at https://github.com/hdore/PB-PSB1_DGR_variation) developed to calculate all possible protein sequence combinations for each VR of each DGR works as follows. The script uses the coordinates of the target protein and VR, and the sequence of the TR to identify the codons (and the positions within each codon) targeted by DGR. It counts all the potential amino acids that can be generated by changing nucleotides at all targeted positions of the codon. To be more accurate for DGR7 locus we used the TR sequence identified in a long-read structural variant (SI Dataset S8).

Annotation of MITE-like sequences

Regions of short direct and inverted repeats at the DGR loci were identified by manual inspection of dot plots and their annotation was refined using Find Repeats with Geneious Prime 2022.1.1 (https://www.geneious.com). These repeats were searched against a database of terminal inverted repeats from the intact IS elements detected with ISEScan using BLASTn with parameters adjusted for short search sequence and to maximize hits covering the entire repeat length (-word_size 7 -gapopen 3 -gapextend 2 -reward 1 -penalty -1). Short inverted repeats matching existing IS-elements were analyzed with RNA Fold (http://rna.tbi.univie.ac.at/cgi-bin/RNAWebSuite/RNAfold.cgi) (26). Stable hairpin-forming inverted repeats were characterized as Miniature Inverted-repeat Transposable Elements (MITE)-like sequences.

Calculation of genome-level nucleotide diversity

The mean nucleotide diversity across *Thiohalocapsa* PB-PSB1's genome was calculated for each of the 184 short-read metagenomes using inStrain v1.8.0 (27), with default parameters except --min_cov 5 and --rarefied_coverage 5. inStrain was run on the bam files generated by bwa-mem (see main Methods). Aggregates with less than 5x mean coverage to PB-PSB1's genome were discarded.

In order to verify whether the diversity was higher within an aggregate that between aggregates in a pond, a selection of aggregates was made: first, aggregates with less than 5x mean coverage were filtered out. As only 8 aggregates had more than 5x mean coverage in pond F, and to allow a fair comparison between ponds, 8 aggregates were then randomly chosen from each pond. To avoid any bias when pooling reads from different aggregates of a given pond, the exact same number of read pairs (260 000 pairs, corresponding to the number of pairs mapped in the aggregate with the lowest coverage) was extracted from each of the selected aggregates. Reads were extracted randomly from the bam files among those mapped (with correct pair mapping) to the PB-PSB1 reference genome. The subsampled reads from each aggregate were then re-mapped to the PB-PSB1 genome with bwa-mem and the resulting bam files were used an input for inStrain (--min_cov 5 and --rarefied_coverage 5), to calculate the nucleotide diversity. The same approach was applied to reads pooled by pond. To compare the diversity of individual aggregates to the diversity of the pool, the rarefied nucleotide diversity was used (inStrain option --rarefied_coverage 5).

Calculation of pink berry volume and correlation to nucleotide diversity

The diameters of aggregates were measured from photographs of aggregates in multiwell plates. The volume was then calculated by approximating the aggregates as spheres, and used as a proxy for the number of cells. We performed regression analyses to test for a linear relationship between the volume of the aggregate and the nucleotide diversity of each VR.

Legends for Datasets S1 to S8

Dataset S1 (separate file).
Coordinates of PB-PSB1 DGR elements. Note that when the element is on the minus strand, the end coordinate is greater than the start coordinate. When multiple VRs are present in a target, they are numbered from the N-terminal end to the C-terminal end. All VR to TR coordinates were verified manually based on VR/TR alignment.

Dataset S2 (separate file).
Calculation of diversification potential for each VR of each DGR target in PB-PSB1. The codons that are targeted by DGR were identified based on the TR/VR alignment (corresponding to positions were the TR has an A). Then the number of potential amino acids (or stop codons) that can be generated by DGR-induced mutation from each codon was calculated using the standard bacterial genetic code. Note that while the codons are identified on the target genes, the codon reference sequence is based on the aligned TR sequence, as the DGR mechanism replaces the whole VR with the TR sequence, including non-variable positions. The table includes the sequence of targeted codons and the positions that are targeted by DGR within each codon. The smaller table on the right-hand side summarizes the results by VR, target and DGR locus. For DGR7 the TR sequence extracted from the long read presenting a structural variant with the intact TR was used for more accuracy.

Dataset S3 (separate file).
Description of all clade 5 DGR-encoding organisms. The metadata available in the IMG Genome database was manually curated, and additional characteristics such as the cell morphology and the multicellular status were added based on the literature cited in the column "Morphology_citation". The IMG accession numbers of the genome and the reverse transcriptase gene are indicated.

Dataset S4 (separate file).
Results of ISEScan detection of insertion sequences (IS) elements. This table corresponds to the raw output from ISEScan. TIR: Terminal Inverted Repeat. Tpase: transposase.

Dataset S5 (separate file).
Single-aggregate metagenomes metadata and accession numbers.

Dataset S6 (separate file).
Annotation of a selection of conflict system-associated domains in clade 5 RTs neighborhood. The first tab indicates the domains that were searched for and their abbreviations used in Fig. 3 and in the main text. These domains were searched for within 20 kb of the DGR clade 5 RTs. For each domain, a separate tab indicates the domain hits in the organisms represented on Fig. 3. The IMG accession number of the genome, the RT gene and the gene with a domain hit are indicated.

Dataset S7 (separate file).
Amino-acid alignment of the vWA domains, including vWA sequences from ternary conflict systems, as used by (28) to build a hmm profile. The hmm profile was used to search for vWA domains in DGR neighborhoods.

Dataset S8 (separate file).
Genbank file containing the MyDGR annotation of a long read presenting a structural variant of *Thiohalocapsa* PB-PSB1's DGR7 locus with an intact template repeat.

Table S1. Distribution and multicellular status of organisms containing more than one clade 5 error-prone reverse transcriptase gene. Cyanobacteria are shaded in green and CPR are shaded in brown. Average phylogenetic distance is the mean of pairwise phylogenetic distance for all clade 5 RT genes in a genome.

Nb of full-length RTs	Average RT Phylogenetic Distance	Number of RTs within each clade 5 subclade							Species	Multicellular or aggregate-associated	IMG Genome ID
		5A	5B	5C	5D	5E	5F	Other			
5	3.45	DGR8	DGR2-4	DGR7-9	DGR3-6				Thiohalocapsa sp. PB-PSB1 (all RTs included	yes	2867970272
4	1.79				1			3	Ca. Magnetomorum sp. HK-1	yes	2648501189
3	2.85	1			2				Ca. Accumulibacter appositus BA-92	yes	2556921088
3	2.75	1						2	Ca. Accumulibacter sp. SK-01 * 2 RTs @ 1 locus	yes	2556921083
3	1.24					3			Achromatium Bin 0	no - highly polyploid	2642422597
3	0.64					3			Calothrix sp. PCC 7103	yes	2507262048
3	0.51					3			Scytonema hofmanni PCC 7110	yes	2551306141
2	4.10	1						1	Ca. Thiodictyon syntrophicum Cad16	yes	2773857920
2	3.81	1			1				Ca. Accumulibacter phosphatis UW-1	yes	CP001715.1
2	3.37	1			1				Verrucomicrobiaceae EBPR_Bin_208	yes	2619618930
2	3.33	1			1				Ca. Accumulibacter sp. SK-11	yes	2556921085
2	3.25			1	1				Ca. Accumulibacter sp. BA-91	yes	2556921087
2	2.74						2		Bdellovibrionaceae NAT178	unknown	2802429364
2	2.54							2	Pelodictyon phaeoclathratiforme BU-1	yes	642555146
2	2.21							2	Thermoflexibacter ruber DSM 9560	yes	2636415974
2	1.93							2	Phaeodactylibacter xiamenensis KD52	yes	2617271238
2	1.69		1			1			Thiomargarita nelsonii bud S10	yes	2600255314
2	1.35						2		Parcubacteria GW2011_GWA2_45_13	unknown	2626541992
2	1.26				2				Viridilinea mediisalina Kir15-3F	yes	2751186036
2	1.06					2			Trichodesmium erythraeum IMS101	yes	637000329
2	1.00					2			Crocosphaera chwakensis CCY0110	yes	640612201
2	0.99				2				Levilinea saccharolytica DSM 16555	yes	2740892510
2	0.95						2		Ca. Woesearchaeota CG10_big_fil_rev_8_21_14_0_10_30_7	unknown	2785511156
2	0.91						2		Ca. Staskawiczbacteria RIFOXYB1_FULL_32_11	unknown	2711768669
2	0.75					2			Nostoc sp. PCC 7120	yes	637000199
2	0.67					2			Merismopedia glauca CCAP 1448/3	yes	2802429465
2	0.65					2			Lake Mendota Epilimnion MEint.metabat.2353	unknown	2582580551
2	0.63					2			Nodularia spumigena CCY9414	yes	2562617131
2	0.63					2			Lake Mendota Epilimnion MEint.metabat.4498	unknown	2582580576
2	0.51					2			Fischerella sp. PCC 9431	yes	2512875027
2	0.48						2		Ca. Pacearchaeota CG10_big_fil_rev_8_21_14_0_10_34_12	unknown	2785510794
2	0.42						2		Armatimonadetes CG06_land_8_20_14_3_00_66_21	unknown	2786546208
2	0.35						2		Ca. Pacearchaeota CG10_big_fil_rev_8_21_14_0_10_30_48	unknown	2785510795
2	0.33					2			Leptolyngbya sp. PCC 7375	yes	2509601039

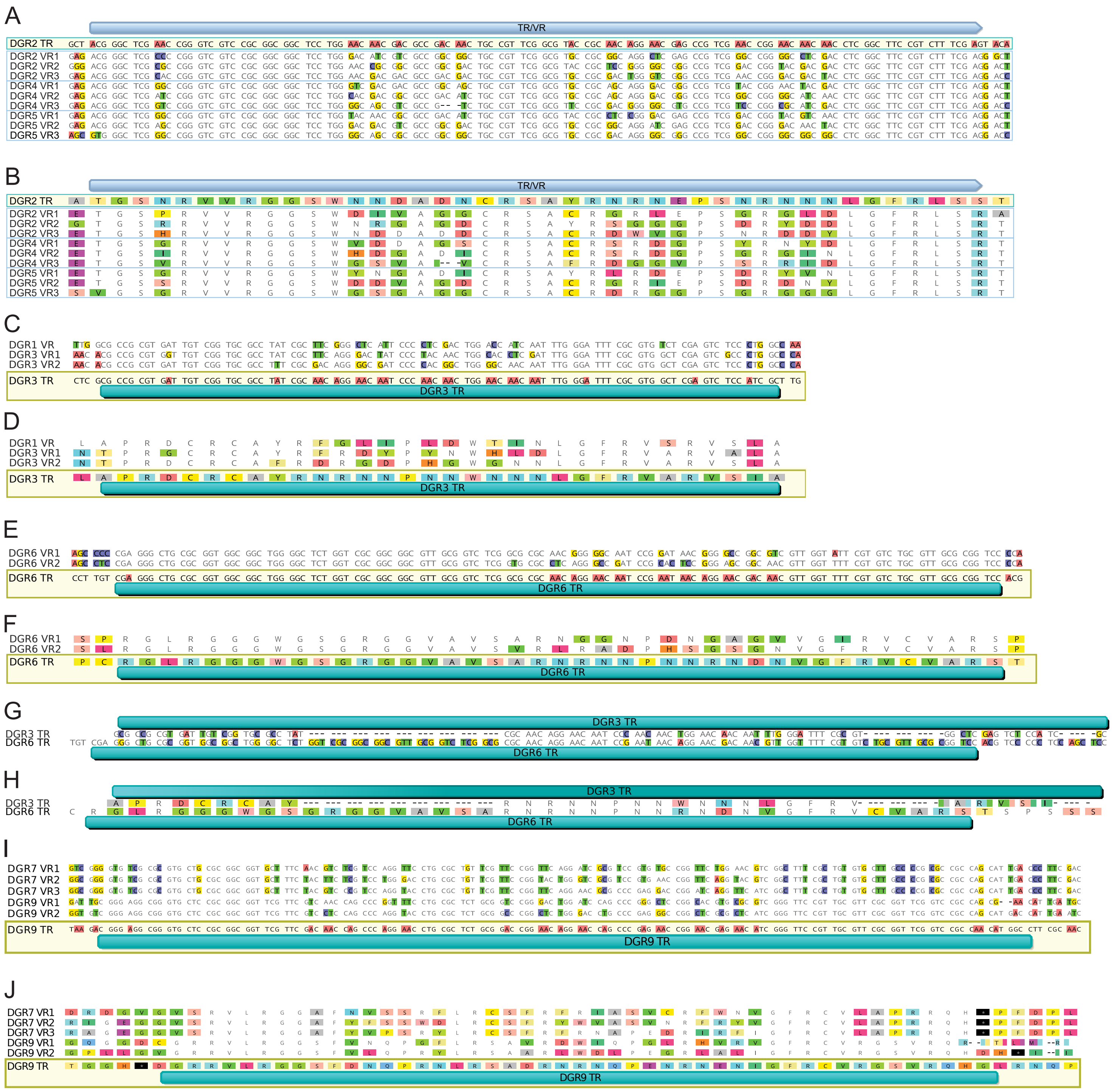

Fig. S1. Alignment of VRs-TR from DGR2-4-5, DGR1-3-6 and DGR7-9.
(**A**) Nucleotide alignment of the TR from DGR2 (top, highlighted in yellow), and the VRs from DGR2/4/5. The predicted TR/VR region is indicated above the sequences, and the positions that are As in the TR are shown in red. Only the mismatches to the TR are colored. (**B**) Amino-acid alignment of the TR from DGR2 and VRs from DGR2/4/5. Although the TR is not translated, the theoretical sequence is shown for comparison to the VRs. (**C**) Nucleotide alignment of the TR of DGR3 and the VRs of DGR1 and DGR3. The location of the predicted TR region is indicated below the sequences, and the positions that are As in the TR are shown in red. Only the mismatches to the TR are colored. (**D**) Amino acid alignment of the TR of DGR3 and the VRs from DGR1 and DGR3. Although the TR is not translated, the theoretical sequence is shown for comparison to the VRs. (**E**) Nucleotide alignment of the TR and VRs of DGR6. The location of the predicted TR region is indicated below the sequences, and the positions that are As in the TR are shown in red. Only the mismatches to the TR are colored. (**F**) Amino acid alignment of the TR and VRs from DGR6. Although the TR is not translated, the theoretical sequence is shown for comparison to the VRs. (**G**) Nucleotide alignment of DGR3 TR and DGR6 TR. Differences between the two sequences are highlighted. (**H**) Amino-acid alignment of DGR3 TR and DGR6 TR. Although the TR is not translated, the theoretical sequence is shown to show the effect of substitutions. (**I**) Nucleotide alignment of the TR from DGR9 (bottom, highlighted in yellow), and the VRs from DGR7/9. The predicted TR/VR region is indicated below the sequences, and the positions that are As in the TR are shown in red. Only the mismatches to the TR are colored. (**J**) Amino-acid alignment of the TR from DGR9 and VRs from DGR7/9. Although the TR is not translated, the theoretical sequence is shown for comparison to the VRs.

A

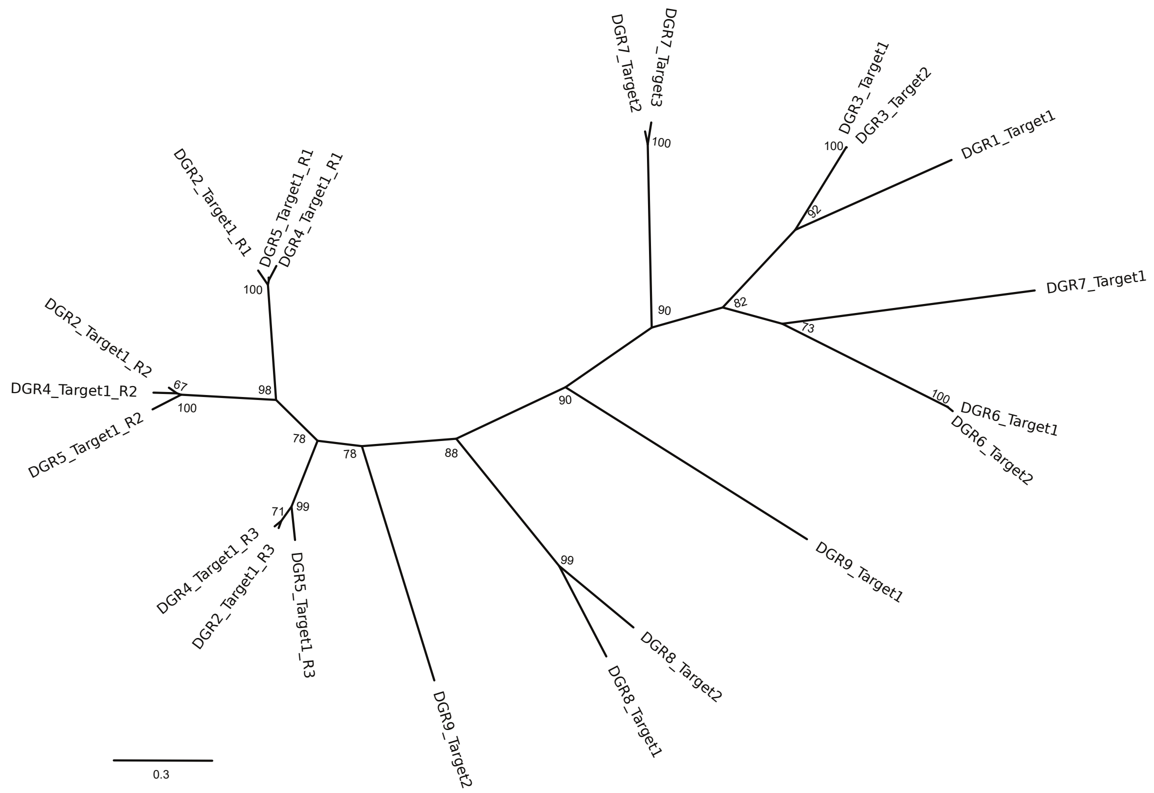

B

	DGR2_Target1_R1	DGR5_Target1_R1	DGR4_Target1_R1	DGR2_Target1_R2	DGR4_Target1_R2	DGR5_Target1_R2	DGR2_Target1_R3	DGR4_Target1_R3	DGR5_Target1_R3	DGR9_Target2	DGR8_Target1	DGR8_Target2	DGR3_Target1	DGR3_Target2	DGR1_Target1	DGR7_Target1	DGR6_Target2	DGR6_Target1	DGR7_Target2	DGR7_Target3	DGR9_Target1
DGR2_Target1_R1		96	70	70	70	63	63	61	48	41	42	40	40	40	43	38	38	42	43	40	40
DGR5_Target1_R1	97		96	70	71	70	63	64	62	48	41	42	39	39	40	43	38	38	42	43	39
DGR4_Target1_R1	96	96		70	70	70	63	64	62	48	40	42	40	40	40	43	37	37	42	43	38
DGR2_Target1_R2	70	70	70		98	94	67	68	65	51	46	49	37	37	38	43	39	39	41	41	44
DGR4_Target1_R2	70	71	70	98		93	66	68	65	52	46	50	38	38	38	43	40	40	40	40	44
DGR5_Target1_R2	70	70	70	94	93		65	67	60	50	44	48	37	37	38	41	40	40	40	40	42
DGR2_Target1_R3	63	63	63	67	66	65		95	85	46	46	45	41	41	39	40	39	39	41	43	38
DGR4_Target1_R3	63	64	64	68	68	67	95		86	46	45	43	40	40	37	39	37	36	40	41	37
DGR5_Target1_R3	61	62	62	65	65	60	85	86		46	46	42	38	38	35	39	35	35	40	41	37
DGR9_Target2	48	48	48	51	52	50	46	46	46		51	53	35	35	33	39	40	40	48	47	43
DGR8_Target1	41	41	40	46	46	44	46	45	46	51		77	33	33	31	38	41	41	41	42	45
DGR8_Target2	42	42	42	49	50	48	45	43	42	53	77		34	34	34	40	43	43	40	42	45
DGR3_Target1	40	39	40	37	38	37	41	40	38	35	33	34		100	59	41	42	42	49	49	33
DGR3_Target2	40	39	40	37	38	37	41	40	38	35	33	34	100		59	41	42	42	49	49	33
DGR1_Target1	40	40	40	38	38	38	39	37	35	33	31	34	59	59		43	47	47	48	48	32
DGR7_Target1	43	43	43	43	43	41	40	39	39	39	38	40	41	41	43		54	54	45	44	37
DGR6_Target2	38	38	37	39	40	40	39	37	35	40	41	43	42	42	47	54		99	54	54	41
DGR6_Target1	38	38	37	39	40	40	39	36	35	40	41	43	42	42	47	54	99		54	54	40
DGR7_Target2	42	42	42	41	40	40	41	40	40	48	41	40	49	49	48	45	54	54		96	42
DGR7_Target3	43	43	43	41	40	40	43	41	41	47	42	42	49	49	48	44	54	54	96		42
DGR9_Target1	40	39	38	44	44	42	38	37	37	43	45	45	33	33	32	37	41	40	42	42	

Fig. S2. Sequence similarity of the C-terminal lectin domains from *Thiohalocapsa* sp. PB-PSB1's 15 DGR target proteins.
(**A**) Maximum likelihood phylogeny of the CLec domains from the 15 target proteins. The variable region of each CLec domain has been removed prior to alignment. Bootstrap support is shown at each node (n=100). Each domain is identified by the DGR locus, target gene name, and for targets with multiple CLec domains they have been numbered from 5' to 3' (R1, R2, R3). (**B**) Amino acid similarity (BLOSUM 62) distance matrix for the CLec domain alignment used for the phylogeny in panel A (the variable region of each CLec domain has been removed prior to alignment).

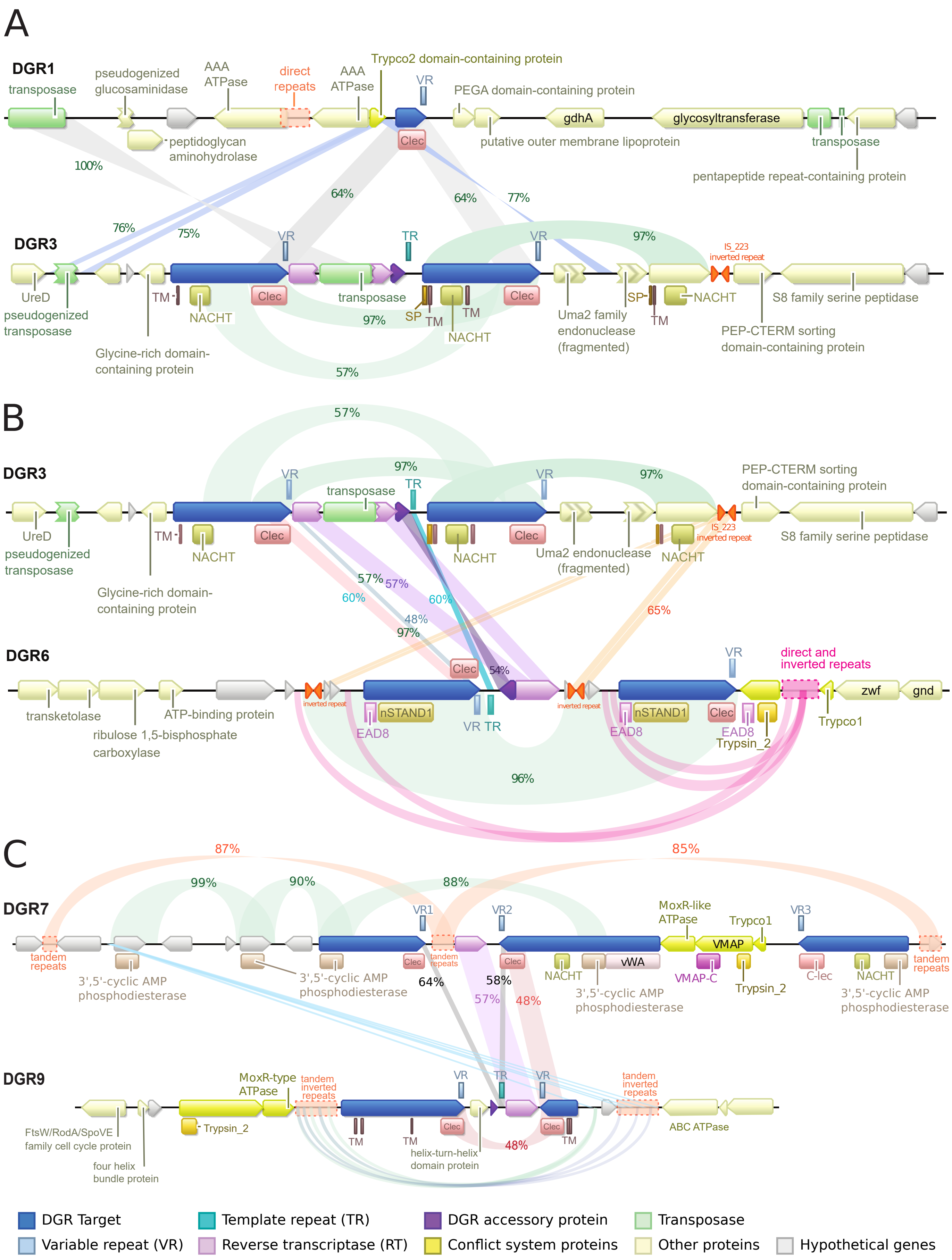

Fig. S3. Comparison of DGR loci 1 and 3 (**A**), 3 and 6 (**B**) and 7 and 9 (**C**). The gene neighborhoods surrounding each DGR locus are shown with regions of similarity highlighted along with the percent nucleotide identity.

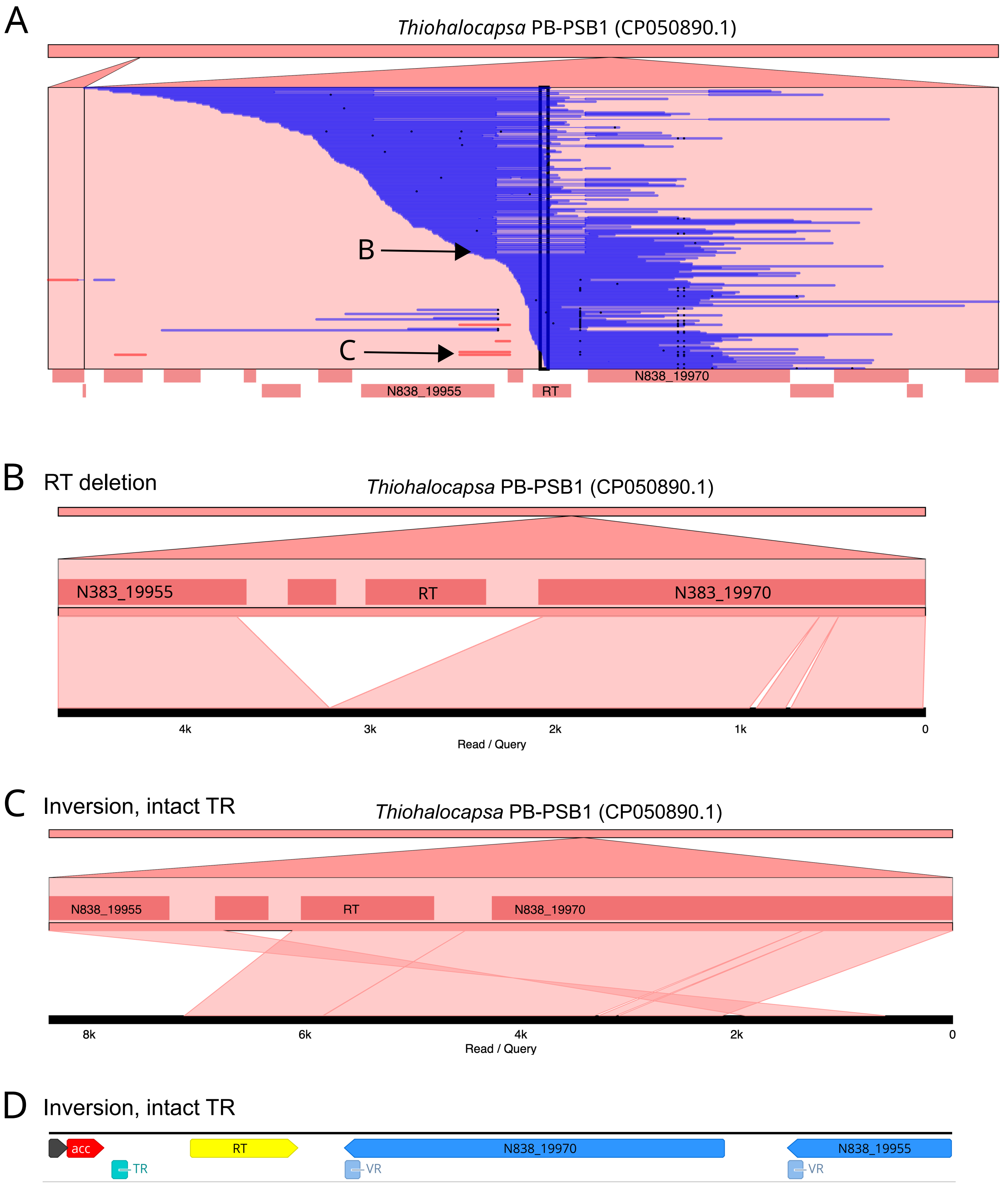

Fig. S4. Structural variants observed at the DGR7 locus.
Structural variants are shown as seen in the long-read alignments to the *Thiohalocapsa* sp. PB-PSB1 reference genome visualized with Genome Ribbon (29). (**A**) Read mappings are shown in blue, with inversions shown in red, short indels shown in black, and longer deletions shown with a thin blue connecting line. Ribbon plots of individual reads show examples of variants with a deleted RT gene (**B**) and variants with inversions and an intact template repeat region (TR) (**C**). Panel (**D**) shows the same read as in panel (**C**) with the intact DGR region annotated using myDGR (30).

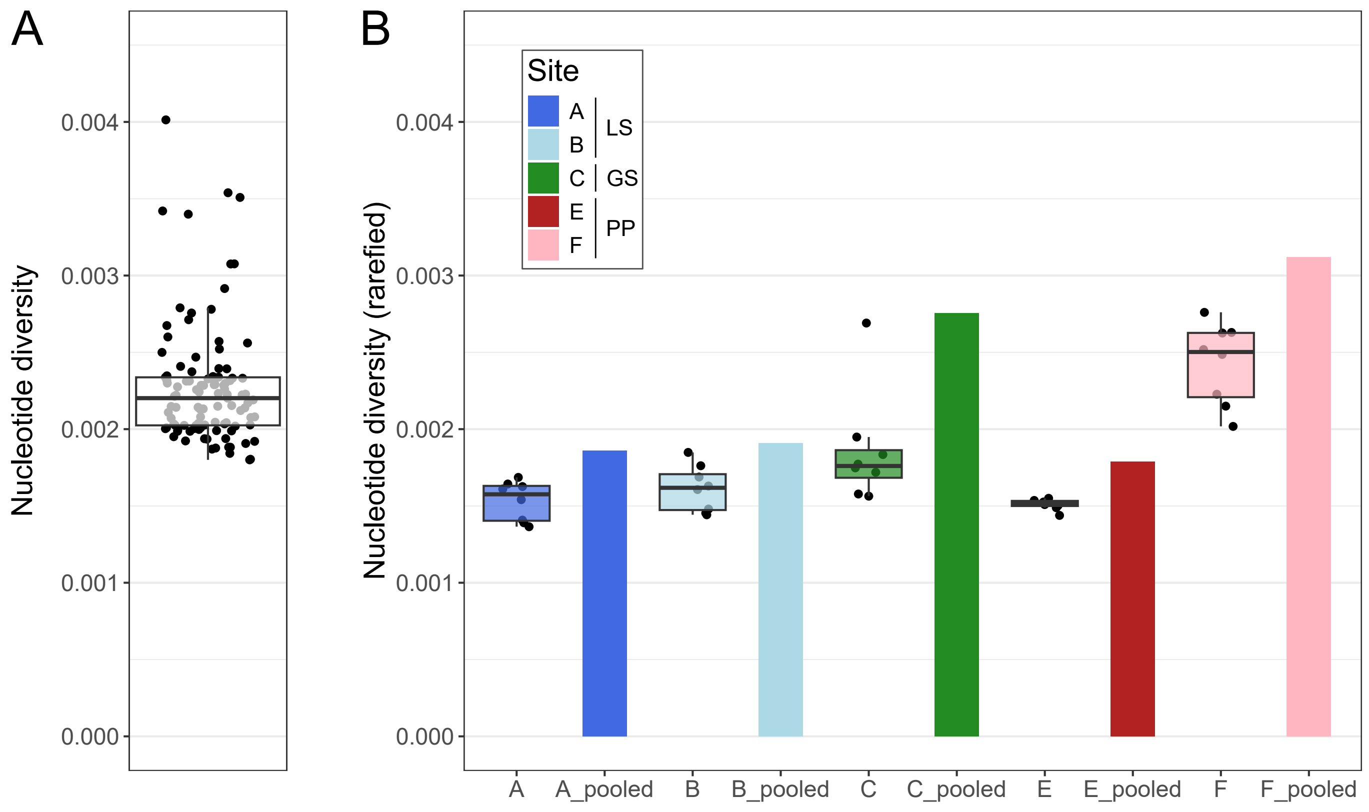

Fig. S5. *Thiohalocapsa* PB-PSB1 nucleotide diversity within and between Pink Berry aggregates.
(**A**) The distribution of whole genome, within-aggregate nucleotide diversity, calculated for every aggregates with more than 5x mean coverage. (**B**) Comparison of within-aggregate and pond-pooled rarefied nucleotide diversity in a selection of aggregates. Eight aggregates where randomly chosen from each pond and subsampled to the exact same number of reads. To allow for a fair comparison, the nucleotide diversity shown in panel B is rarefied to 5x, which explains the lower values compared to panel A.

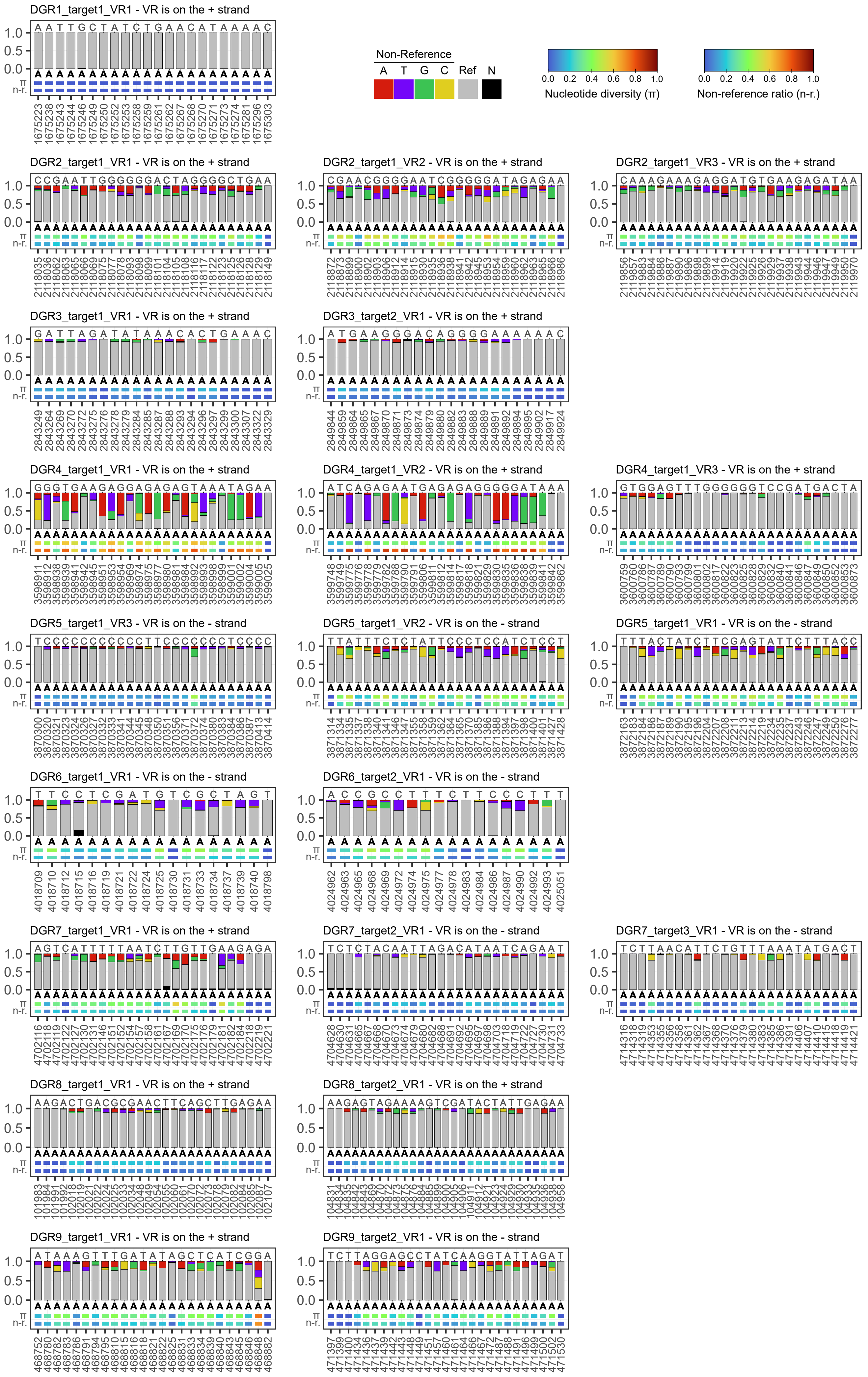

Fig. S6. *In situ* diversification of VRs of all DGR loci in LS01_001 pink berry aggregate from long-reads metagenomics data.

Only positions that correspond to an A in the TR are shown. Bar plots indicate the proportion of A, T, C and G at each position, colored if they differ from the reference. Letters above bars indicate the VR sequence in the reference genome, while letters below bars indicate the reference sequence of the TR. Bottom rows show the nucleotide diversity (π) and proportion of non-reference alleles (n-r.) at each position. Ref.: Reference nucleotide. N: unknown nucleotide.

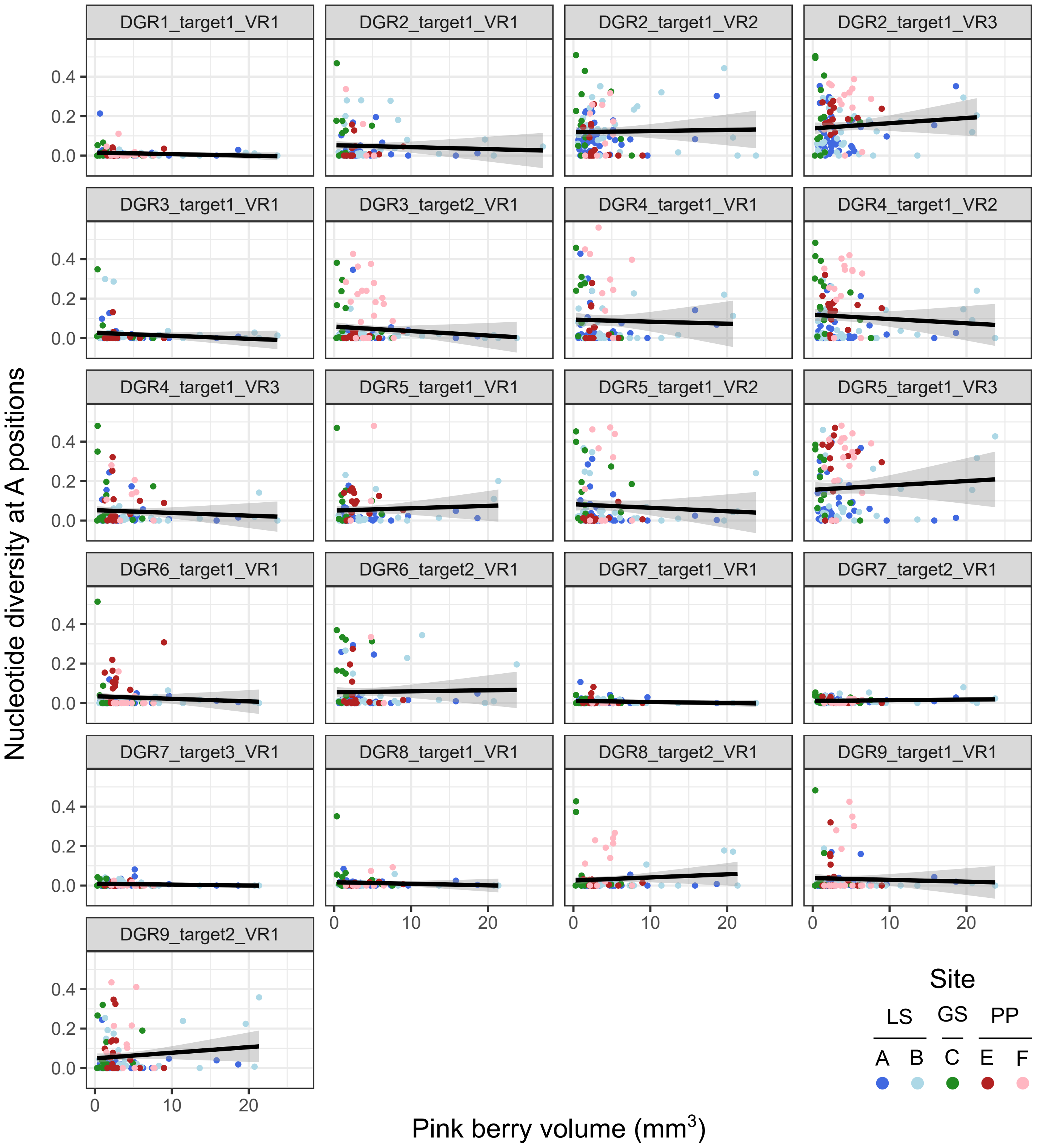

Fig. S7. Correlation between the volume of aggregates and the within-aggregate nucleotide diversity of *Thiohalocapsa* PB-PSB1 DGRs.
The volume of each aggregate was calculated from its diameter by considering the aggregate as a sphere. For each DGR VR, the nucleotide diversity was averaged over all positions that correspond to an A in the TR, which are the targets of DGR diversification. The solid line corresponds to a linear regression, with the grey shading indicating the 95% confidence interval. All regressions had a *p*-value > 0.05.

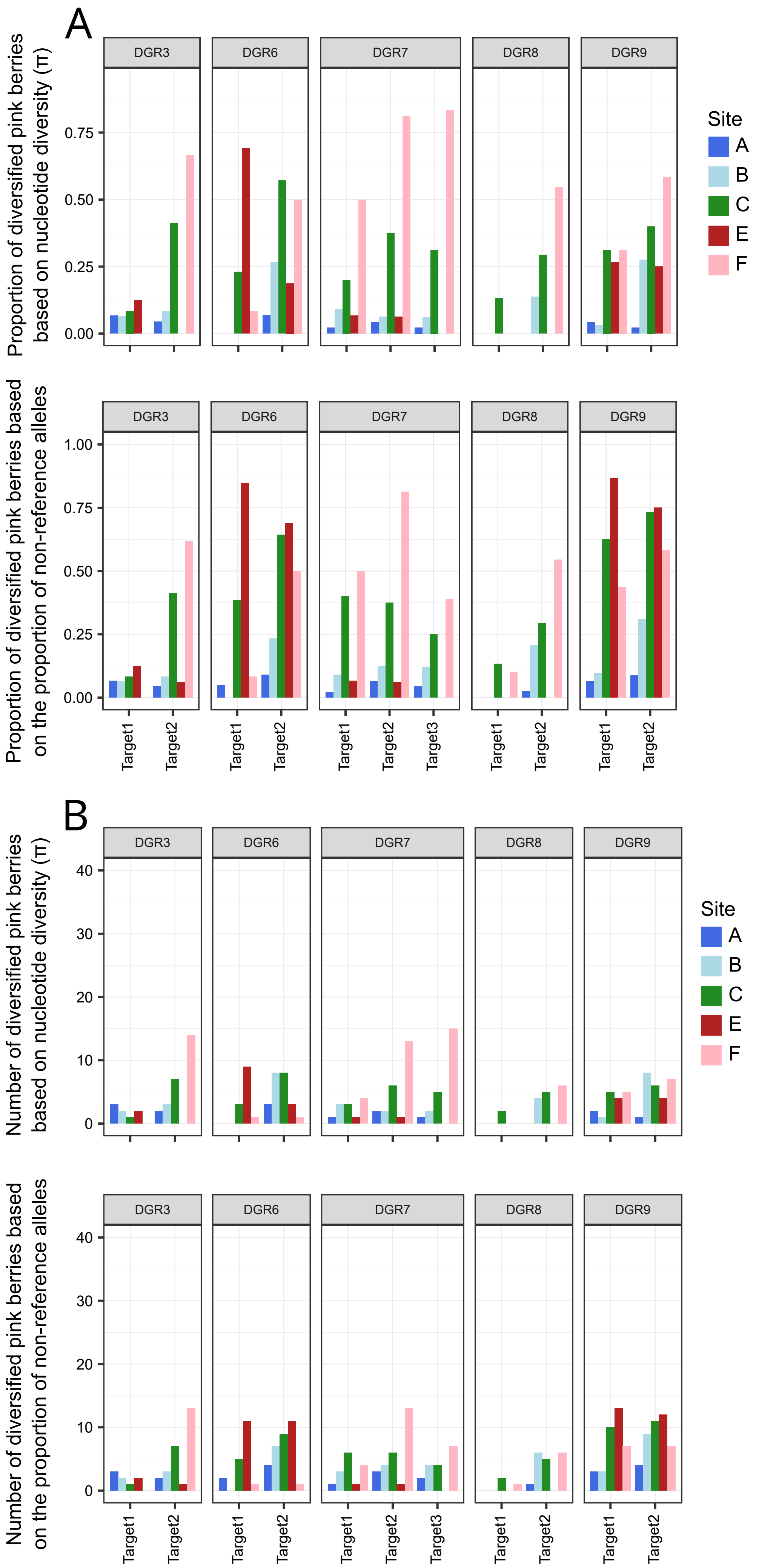

Fig. S8. Proportion (**A**) and number (**B**) of aggregates showing diversification for each DGR target gene at each sampling site. A DGR target was considered diversified if at least one of its VRs showed diversification at positions targeted by the DGR mechanism based on the nucleotide diversity (upper panel) or the proportion of non-reference alleles (lower panel). Only DGR loci with multiple targets are represented. Colors correspond to sampling sites, with blue shades corresponding to Little Sippewissett salt marsh (LS), green to Great Sippewissett (GS) and red shades to Penzance Point (PP).

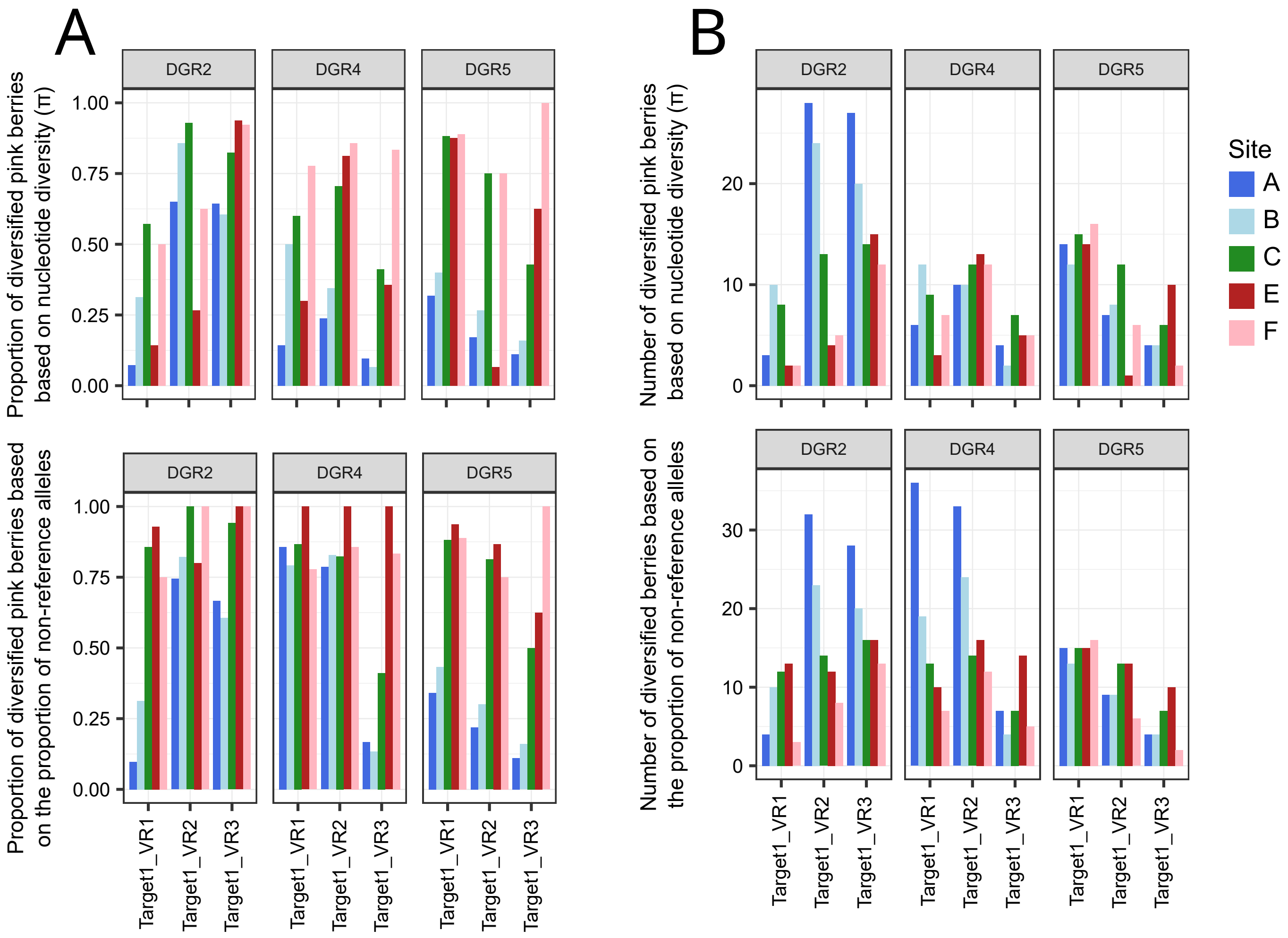

Fig. S9. Proportion (**A**) and number (**B**) of aggregates showing diversification for each VR of each DGR target gene at each sampling site. Nucleotide diversity (upper panel) or the proportion of non-reference alleles (lower panel) were used to determine if a VR was diversified. Only DGR targets with multiple VRs are represented. Colors correspond to sampling sites, with blue shades corresponding to Little Sippewissett salt marsh (LS), green to Great Sippewissett salt marsh (GS) and red shades to Penzance Point salt marsh (PP).

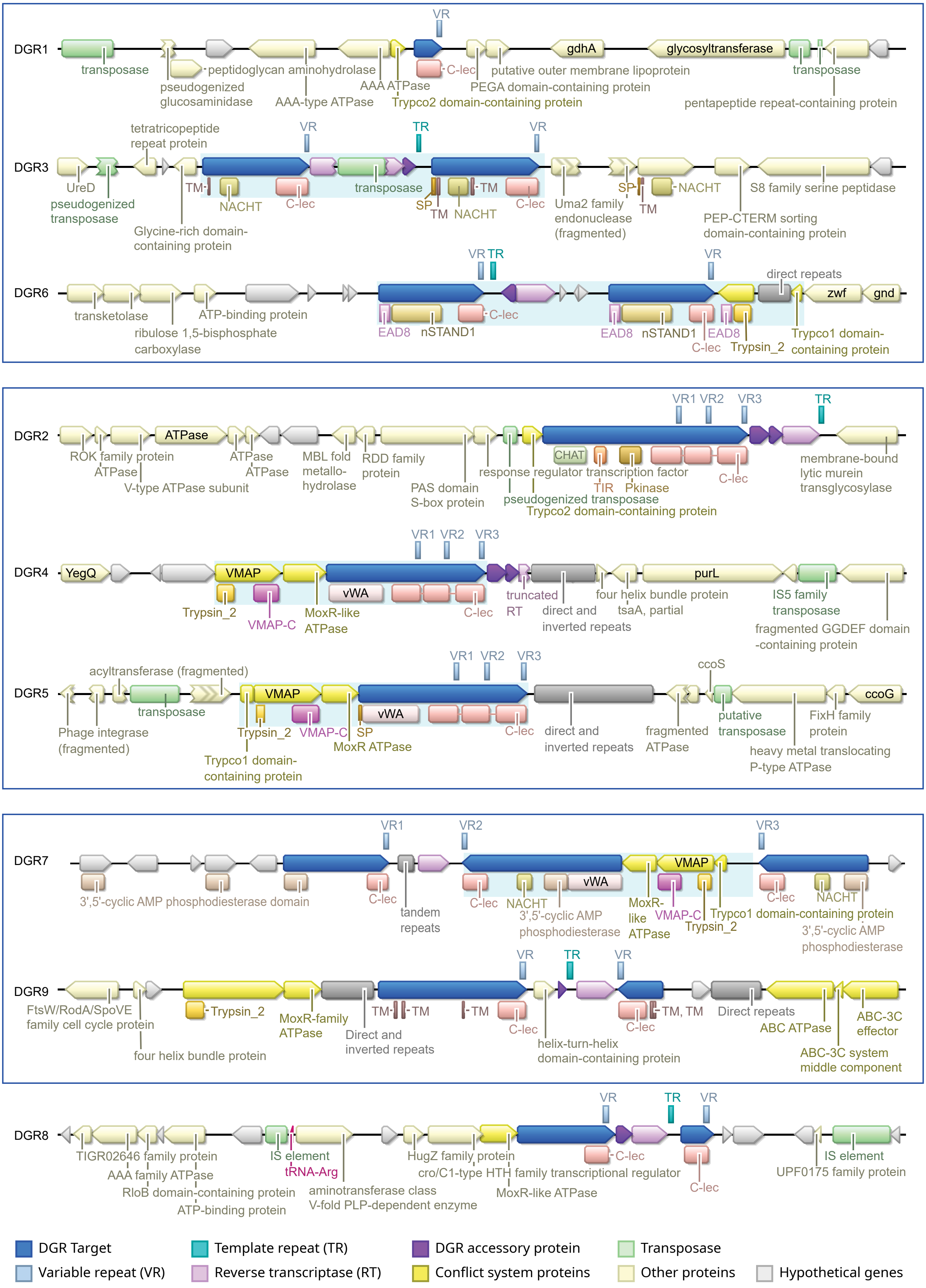

Fig. S10. Functional annotation of genes surrounding all *Thiohalocapsa* PB-PSB1 DGR loci.
The light blue shaded areas indicate gene architectures described as putative conflict systems. SP: signal peptide, TM: transmembrane domain.

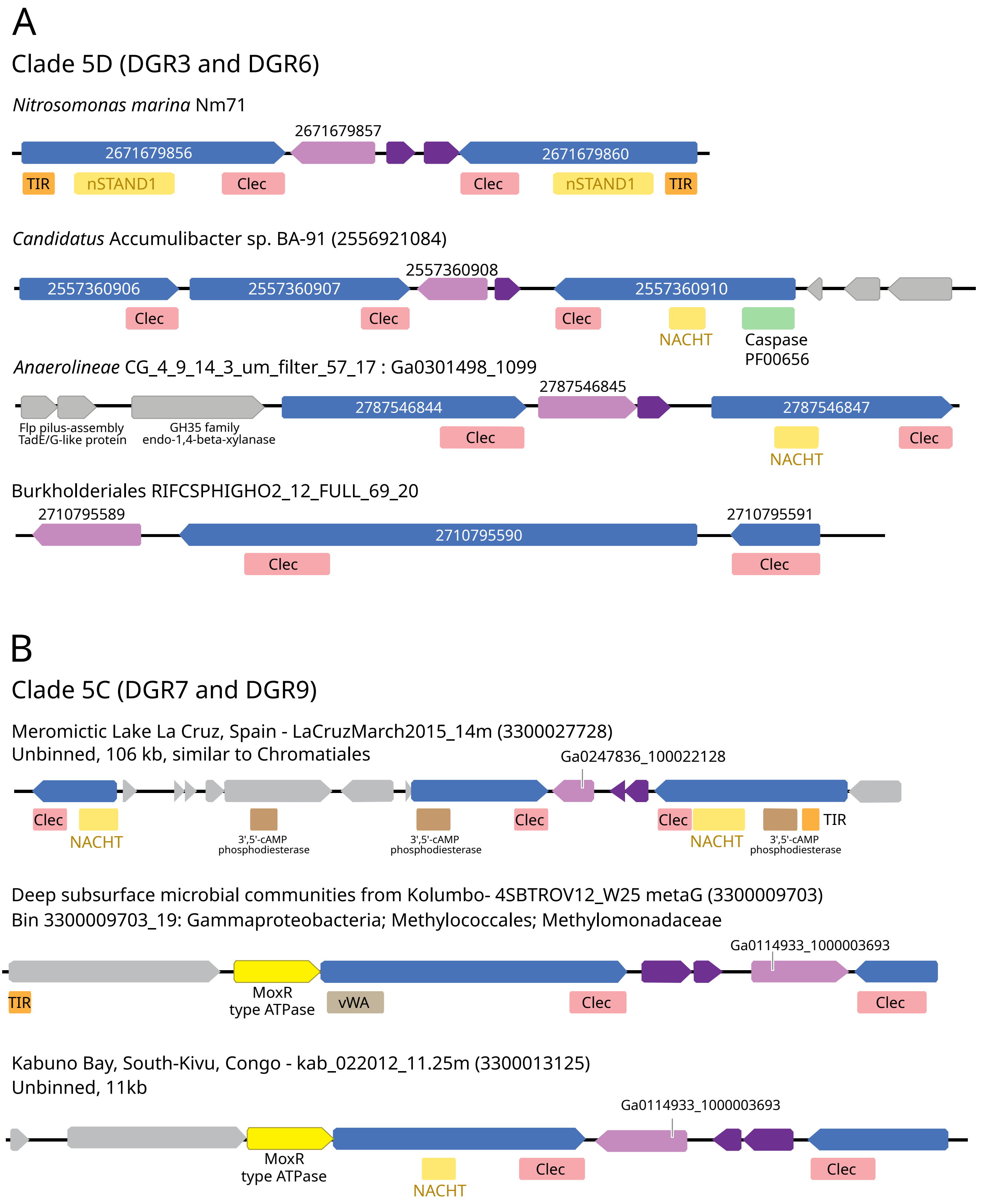

Fig. S11. Examples of clade 5 DGR loci with a gene organization similar to *Thiohalocapsa* PB-PSB1 DGRs. (**A**) DGR loci in clade 5D. (**B**) DGR loci in clade 5C. These examples correspond to metagenomic contigs. (**C**) DGR loci in clade 5B. The last example corresponds to a metagenomics contig. (**D**) DGR loci in clade 5A.

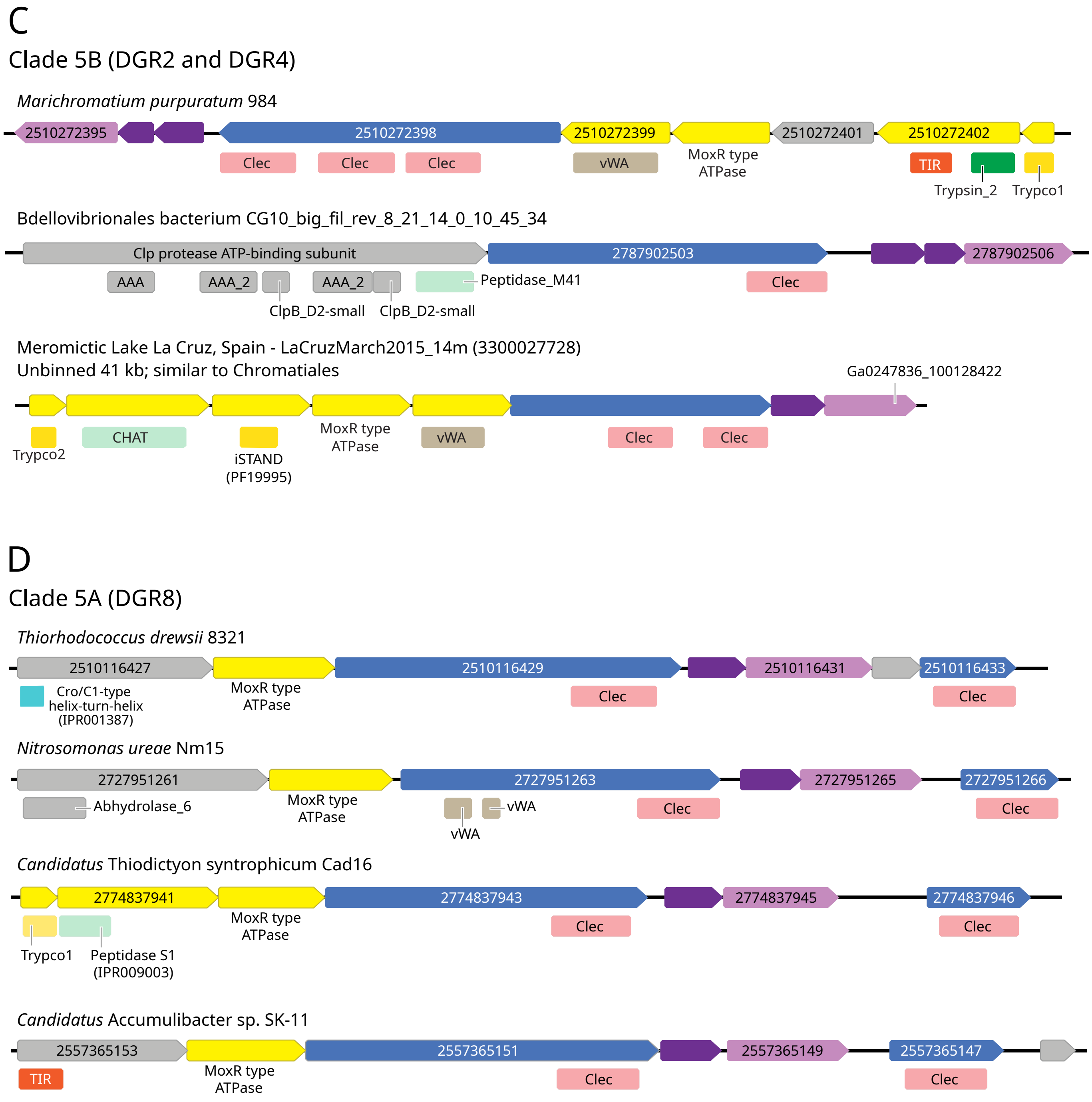

Fig. S11. Examples of clade 5 DGR loci with a gene organization similar to *Thiohalocapsa* PB-PSB1 DGRs. (**A**) DGR loci in clade 5D. (**B**) DGR loci in clade 5C. These examples correspond to metagenomic contigs. (**C**) DGR loci in clade 5B. The last example corresponds to a metagenomics contig. (**D**) DGR loci in clade 5A.

SI References

1. M. Liu, *et al.*, Reverse Transcriptase-Mediated Tropism Switching in Bordetella Bacteriophage. *Science* **295**, 2091–2094 (2002).
2. S. A. McMahon, *et al.*, The C-type lectin fold as an evolutionary solution for massive sequence variation. *Nat Struct Mol Biol* **12**, 886–892 (2005).
3. B. Medhekar, J. F. Miller, Diversity-generating retroelements. *Current Opinion in Microbiology* **10**, 388–395 (2007).
4. B. G. Paul, *et al.*, Targeted diversity generation by intraterrestrial archaea and archaeal viruses. *Nat Commun* **6**, 6585 (2015).
5. B. R. Macadangdang, S. K. Makanani, J. F. Miller, Accelerated Evolution by Diversity-Generating Retroelements. *Annual Review of Microbiology* **76**, 389–411 (2022).
6. S. Roux, *et al.*, Ecology and molecular targets of hypermutation in the global microbiome. *Nat Commun* **12**, 3076 (2021).
7. T. Yamada, *et al.*, Anaerolinea thermolimosa sp. nov., Levilinea saccharolytica gen. nov., sp. nov. and Leptolinea tardivitalis gen. nov., sp. nov., novel filamentous anaerobes, and description of the new classes Anaerolineae classis nov. and Caldilineae classis nov. in the bacterial phylum Chloroflexi. *International Journal of Systematic and Evolutionary Microbiology* **56**, 1331–1340 (2006).
8. V. M. Gorlenko, *et al.*, Candidatus ‘Chloroploca asiatica’ gen. nov., sp. nov., a new mesophilic filamentous anoxygenic phototrophic bacterium. *Microbiology* **83**, 838–848 (2014).
9. D. S. Grouzdev, M. S. Rysina, I. A. Bryantseva, V. M. Gorlenko, V. A. Gaisin, Draft genome sequences of ‘Candidatus Chloroploca asiatica’ and ‘Candidatus Viridilinea mediisalina’, candidate representatives of the Chloroflexales order: phylogenetic and taxonomic implications. *Standards in Genomic Sciences* **13**, 24 (2018).
10. V. Ivanov, O. Stabnikova, P. Sihanonth, P. Menasveta, Aggregation of ammonia-oxidizing bacteria in microbial biofilm on oyster shell surface. *World J Microbiol Biotechnol* **22**, 807–812 (2006).
11. B. U. Foesel, *et al.*, Nitrosomonas Nm143-like ammonia oxidizers and Nitrospira marina-like nitrite oxidizers dominate the nitrifier community in a marine aquaculture biofilm. *FEMS Microbiology Ecology* **63**, 192–204 (2008).
12. V. J. R. Kumar, V. Joseph, R. Vijai, R. Philip, I. S. B. Singh, Nitrification in a packed bed bioreactor integrated into a marine recirculating maturation system under different substrate concentrations and flow rates. *Journal of Chemical Technology & Biotechnology* **86**, 790–797 (2011).
13. J. F. Imhoff, N. Pfennig, Thioflavicoccus mobilis gen. nov., sp. nov., a novel purple sulfur bacterium with bacteriochlorophyll b. *International Journal of Systematic and Evolutionary Microbiology* **51**, 105–110 (2001).
14. S. Kolinko, M. Richter, F.-O. Glöckner, A. Brachmann, D. Schüler, Single-cell genomics reveals potential for magnetite and greigite biomineralization in an uncultivated multicellular magnetotactic prokaryote. *Environmental Microbiology Reports* **6**, 524–531 (2014).
15. A. Zaar, G. Fuchs, J. R. Golecki, J. Overmann, A new purple sulfur bacterium isolated from a littoral microbial mat, Thiorhodococcus drewsii sp. nov. *Arch Microbiol* **179**, 174–183 (2003).
16. S. Peduzzi, M. Tonolla, D. Hahn, Isolation and characterization of aggregate-forming sulfate-reducing and purple sulfur bacteria from the chemocline of meromictic Lake Cadagno, Switzerland. *FEMS Microbiology Ecology* **45**, 29–37 (2003).
17. V. M. Gorlenko, A new phototrophic green sulphur bacterium. Prosthecochloris aestuarii nov. gen. nov. spec. *Z Allg Mikrobiol* **10**, 147–149 (1970).
18. J. F. Imhoff, H. G. Trüper, Chromatium purpuratum, sp. nov., a new species of the Chromatiaceae. *Zentralblatt für Bakteriologie: I. Abt. Originale C: Allgemeine, angewandte und ökologische Mikrobiologie* **1**, 61–69 (1980).
19. X. A. Walter, *et al.*, Phototrophic Fe(II)-oxidation in the chemocline of a ferruginous meromictic lake. *Frontiers in Microbiology* **5** (2014).
20. C. Riquelme, *et al.*, Actinobacterial Diversity in Volcanic Caves and Associated Geomicrobiological Interactions. *Frontiers in Microbiology* **6** (2015).
21. B. E. Flood, *et al.*, Single-Cell (Meta-)Genomics of a Dimorphic Candidatus Thiomargarita nelsonii Reveals Genomic Plasticity. *Frontiers in Microbiology* **7** (2016).

22. A. J. Probst, *et al.*, Differential depth distribution of microbial function and putative symbionts through sediment-hosted aquifers in the deep terrestrial subsurface. *Nat Microbiol* **3**, 328–336 (2018).
23. J.-R. Arseneau, R. Steeves, M. Laflamme, Modified low-salt CTAB extraction of high-quality DNA from contaminant-rich tissues. *Molecular Ecology Resources* **17**, 686–693 (2017).
24. K. Wilson, Preparation of Genomic DNA from Bacteria. *Current Protocols in Molecular Biology* **56**, 2.4.1-2.4.5 (2001).
25. Z. Xie, H. Tang, ISEScan: automated identification of insertion sequence elements in prokaryotic genomes. *Bioinformatics (Oxford, England)* **33**, 3340–3347 (2017).
26. A. R. Gruber, R. Lorenz, S. H. Bernhart, R. Neuböck, I. L. Hofacker, The Vienna RNA Websuite. *Nucleic Acids Research* **36**, W70–W74 (2008).
27. M. R. Olm, *et al.*, inStrain profiles population microdiversity from metagenomic data and sensitively detects shared microbial strains. *Nat Biotechnol* **39**, 727–736 (2021).
28. G. Kaur, A. M. Burroughs, L. M. Iyer, L. Aravind, Highly regulated, diversifying NTP-dependent biological conflict systems with implications for the emergence of multicellularity. *eLife* **9**, e52696 (2020).
29. M. Nattestad, R. Aboukhalil, C.-S. Chin, M. C. Schatz, Ribbon: intuitive visualization for complex genomic variation. *Bioinformatics* **37**, 413–415 (2021).
30. F. Sharifi, Y. Ye, MyDGR: a server for identification and characterization of diversity-generating retroelements. *Nucleic Acids Research* **47**, W289–W294 (2019).
